# Supplementary material for: Cannabinoid non-cannabidiol site modulation of TRPV2 structure and function
Source: Nat Commun. 2022 Dec 5;13:7483. doi: 10.1038/s41467-022-35163-y (PMC9722916; doi:10.1038/s41467-022-35163-y)
Supplement: Supplementary file 1 — Supplementary Information [file 41467_2022_35163_MOESM1_ESM.pdf]

Supplementary Information

Cannabinoid non-cannabidiol site modulation of TRPV2 structure and function

Liying Zhang[1,2,3#], Charlotte Simonsen[1,#], Lucie Zimova[4], Kaituo Wang[3], Lavanya Moparthi[5,6], Rachelle Gaudet[7], Maria Ekoff[8], Gunnar Nilsson[8], Ute A. Hellmich[9,10], Viktorie Vlachova[4], Pontus Gourdon[2,3,*] and Peter M. Zygmunt[1,*]

[1]Department of Clinical Sciences Malmö, Lund University, Malmö, Sweden.
[2]Department of Experimental Medical Science, Lund University, Lund, Sweden.
[3]Department of Biomedical Sciences, University of Copenhagen, Copenhagen, Denmark.
[4]Department of Cellular Neurophysiology, Institute of Physiology, Czech Academy of Sciences, Prague, Czech Republic.
[5]Wallenberg Centre for Molecular Medicine, Linköping University, Linköping, Sweden.
[6]Department of Biomedical and Clinical Sciences, Linköping University, Linköping, Sweden.
[7]Department of Molecular and Cellular Biology, Harvard University, Cambridge, USA.
[8]Division Immunology and Allergy Unit, Department of Medicine Solna, Karolinska Institutet, Karolinska University Hospital, Solna, Sweden.
[9]Faculty of Chemistry and Earth Sciences, Institute of Organic Chemistry and Macromolecular Chemistry and Cluster of Excellence "Balance of the Microverse", Friedrich Schiller University Jena, Jena, Germany.
[10]Center for Biomolecular Magnetic Resonance, Goethe-University, Frankfurt/Main, Germany.

[*]Correspondence: pontus.gourdon@med.lu.se or peter.zygmunt@med.lu.se

Supplementary Figures 1-25
Supplementary Tables 1 and 2

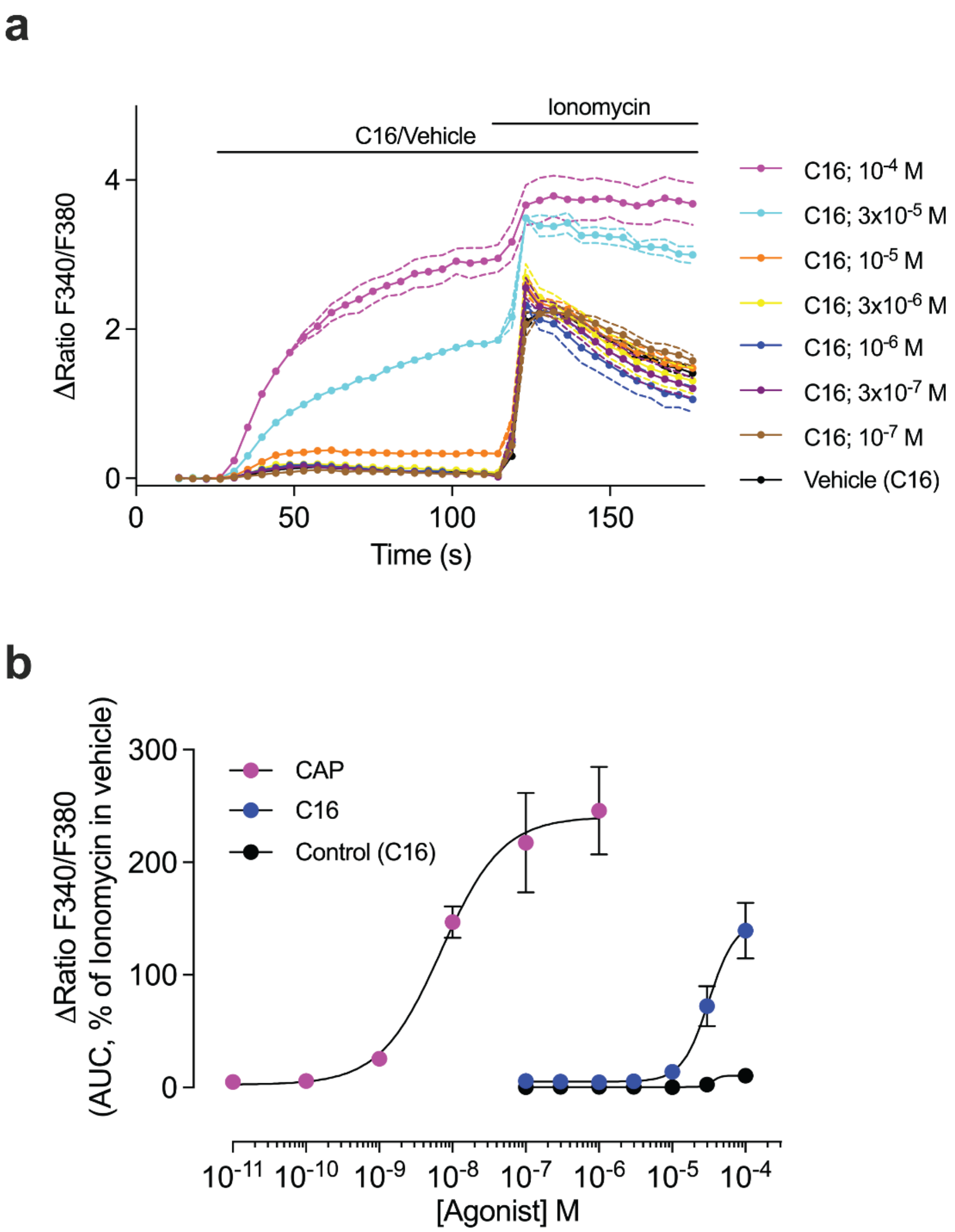

Supplementary Figure 1. C16 activates human TRPV1. a Average $[Ca^{2+}]_i$ responses to different concentrations of C16 (or vehicle) followed by ionomycin (1 μM) in HEK293Trex human TRPV1 inducible cells loaded with the fluorescent calcium indicator Fura-2. Separate experiments with the prototypic TRPV1 agonist capsaicin (CAP) were performed in the same manner. **b** Concentration-dependent changes of $[Ca^{2+}]_i$ triggered by C16 or CAP in HEK293Trex cells expressing human TRPV1 or untransfected HEK293Trex cells (control). Data are shown as means ± SEM from four (C16), three (CAP) or two (control) independent experiments each performed in triplicate. As shown by traces the vehicle for C16 and CAP had no effect (**a**). The responses were analyzed as area under the curve (AUC) for C16 and CAP, and calculated as a percentage of the ionomycin response (AUC) in the presence of the vehicle. Source data are provided as a Source Data file.

a

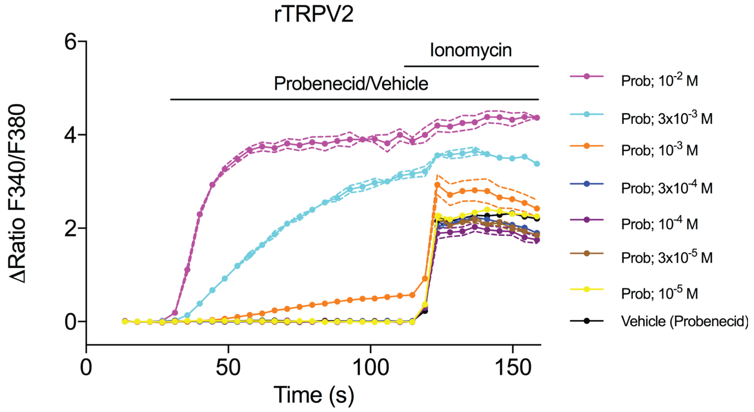

b

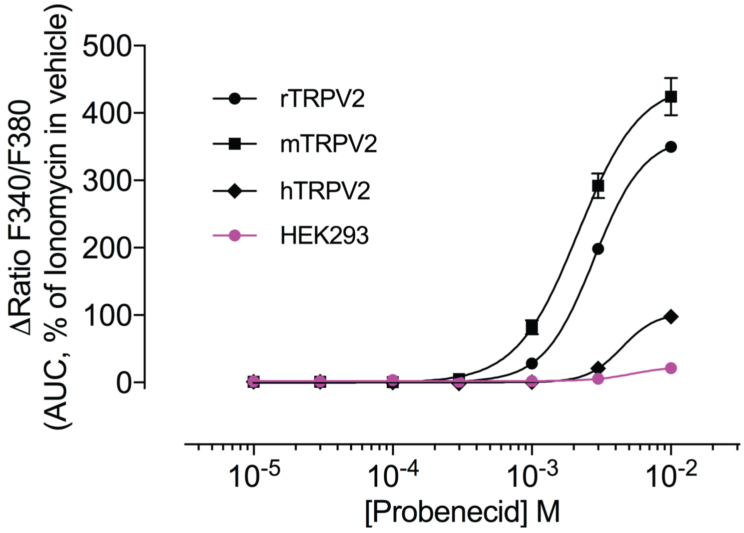

Supplementary Figure 2. Probenecid activates rat, mouse and human TRPV2. **a** Average $[Ca^{2+}]_i$ responses to different concentrations of probenecid (Prob) followed by ionomycin (1 μM) in HEK293T cells heterologously expressing rat TRPV2 and loaded with the fluorescent calcium indicator Fura-2. The same procedure was used in studies on mouse and human TRPV2. **b** Concentration-dependent changes of $[Ca^{2+}]_i$ evoked by probenecid in HEK293T cells expressing either rat, mouse or human TRPV2, but not in untransfected cells. Data are shown as means ± SEM from three independent experiments, each performed in triplicate. As shown by traces the vehicle for probenecid had no effect (**a**). The responses were analyzed as area under the curve (AUC) for probenecid, and calculated as a percentage of the ionomycin response (AUC) in the presence of the probenecid vehicle. Source data are provided as a Source Data file.

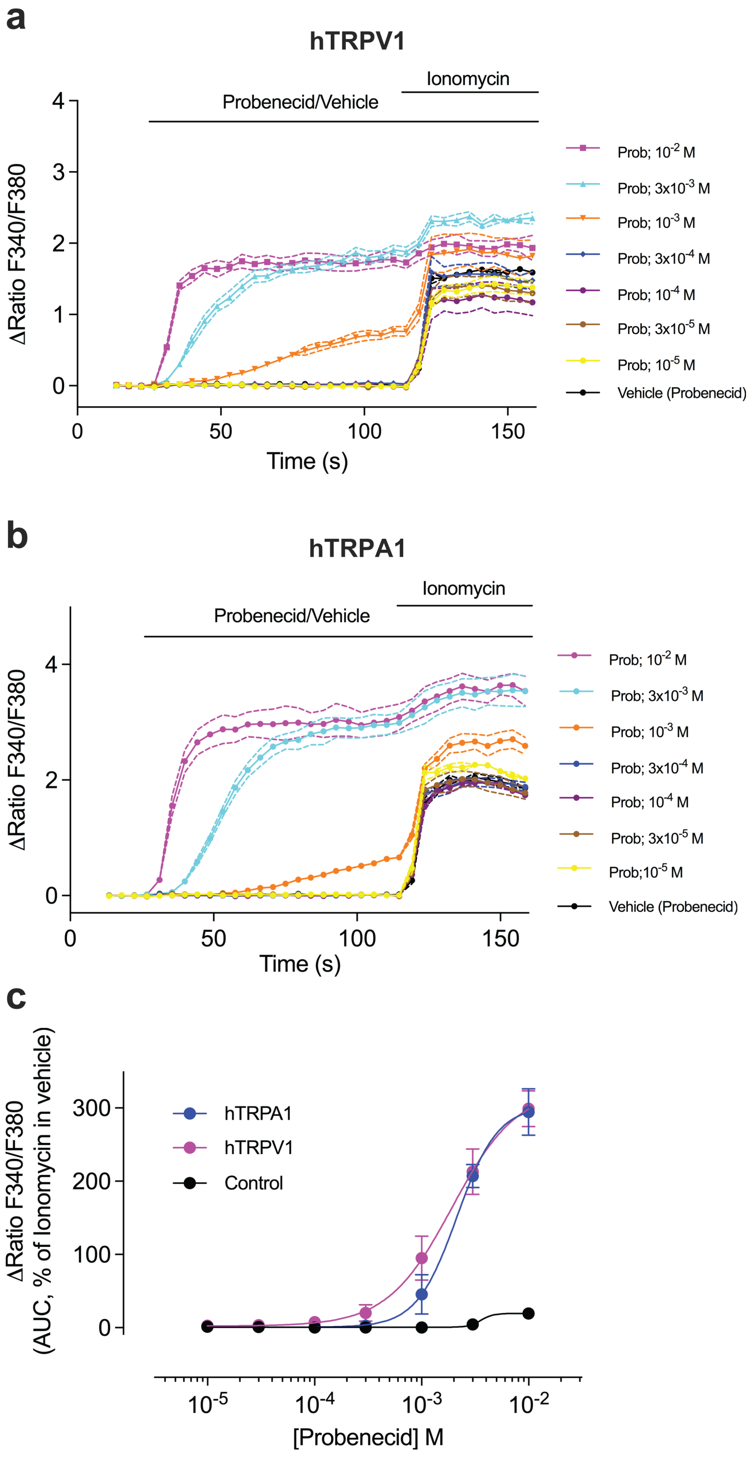

Supplementary Figure 3. Probenecid activates human TRPV1 and TRPA1. **a, b** Average $[Ca^{2+}]_i$ responses to different concentrations of probenecid (Prob; or vehicle) followed by ionomycin (1 µM) in HEK293Trex human TRPV1 (**a**) or TRPA1 (**b**) inducible cells loaded with the fluorescent calcium indicator Fura-2. **c** Concentration-dependent changes of $[Ca^{2+}]_i$ evoked by probenecid in HEK293Trex cells expressing human TRPA1, TRPV1 or untransfected HEK293Trex cells (control). Data are shown as means ± SEM from six (TRPV1), four (TRPA1) or five (control) independent experiments each performed in triplicate. As shown by traces the vehicle for probenecid had no effect (**a**). The responses were analyzed as area under the curve (AUC) for probenecid, and calculated as a percentage of the ionomycin response (AUC) in the presence of the probenecid vehicle. Source data are provided as a Source Data file.

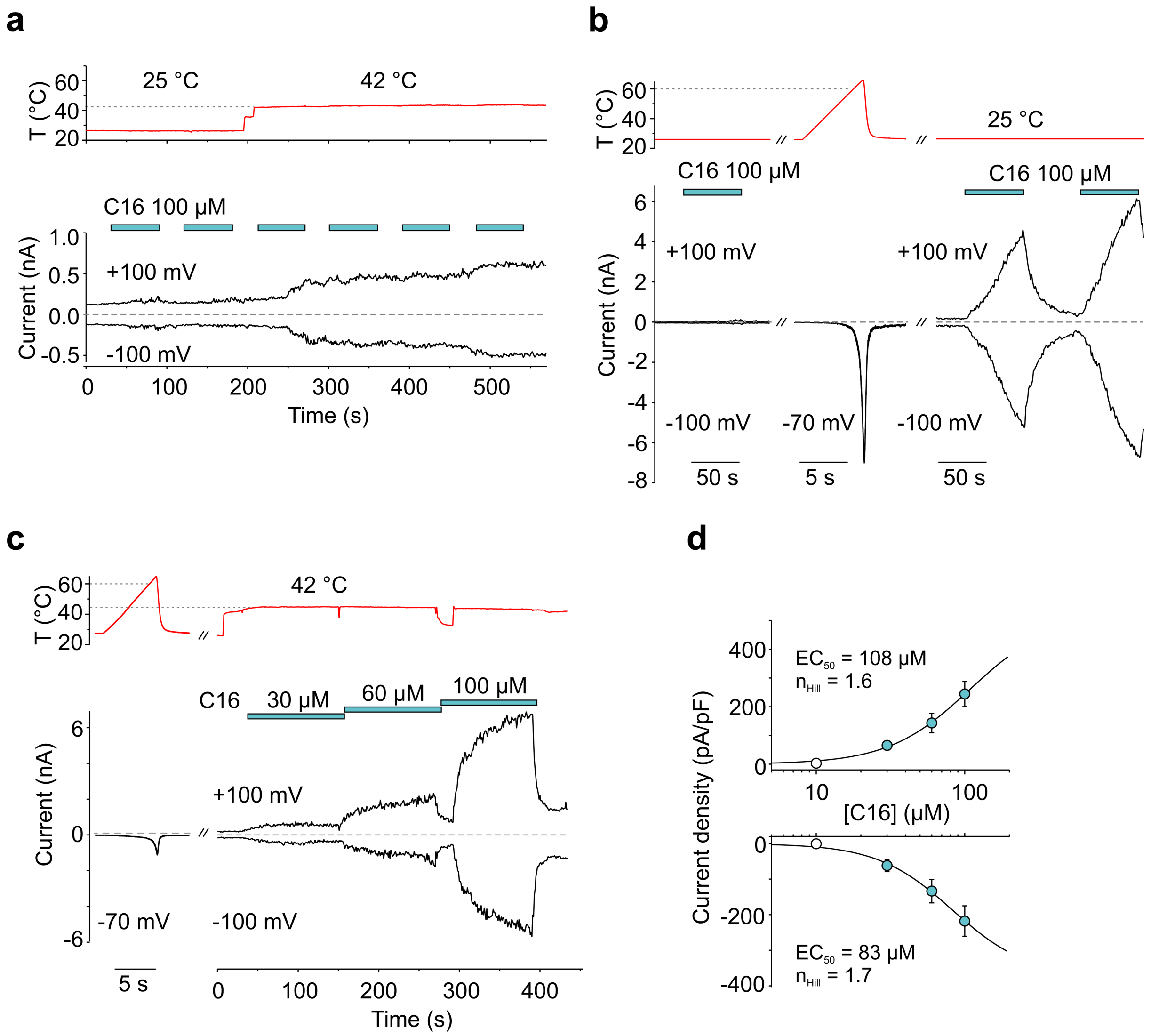

Supplementary Figure 4. Temperature-dependent effects of C16 on rat TRPV2 currents in HEK293T cells. a Representative whole-cell TRPV2 currents elicited by C16 using voltage ramps from -100 mV to +100 mV (0.4 V/s delivered every 1 s from a holding potential of 0 mV), at the temperatures indicated above. C16 100 µM (marked as cyan horizontal bars) was applied repeatedly to the cell at temperatures indicated above. **b** C16 in combination with voltage ramps only barely activates TRPV2 at 25 °C, whereas a prior heat ramp dramatically sensitized subsequent responses to C16. **c** Concentration-dependent effects of C16 on TRPV2 under optimized conditions for obtaining steady-state responses. Whole-cell currents induced by C16 (marked as cyan horizontal bars) applied sequentially at concentrations 30, 60 and 100 µM, recorded at 42 °C. **d** Average current densities produced by C16 at +100 and -100 mV, measured at 42 °C, were fitted by concentration-response curves. Data are shown as means ± SEM from 13 (30 µM), 11 (60 µM) and 11 (100 µM) independent experiments recorded in extra- and intracellular Ca^{2+}-free solutions. Shown is also the current density from one recording at 10 µM. Source data are provided as a Source Data file.

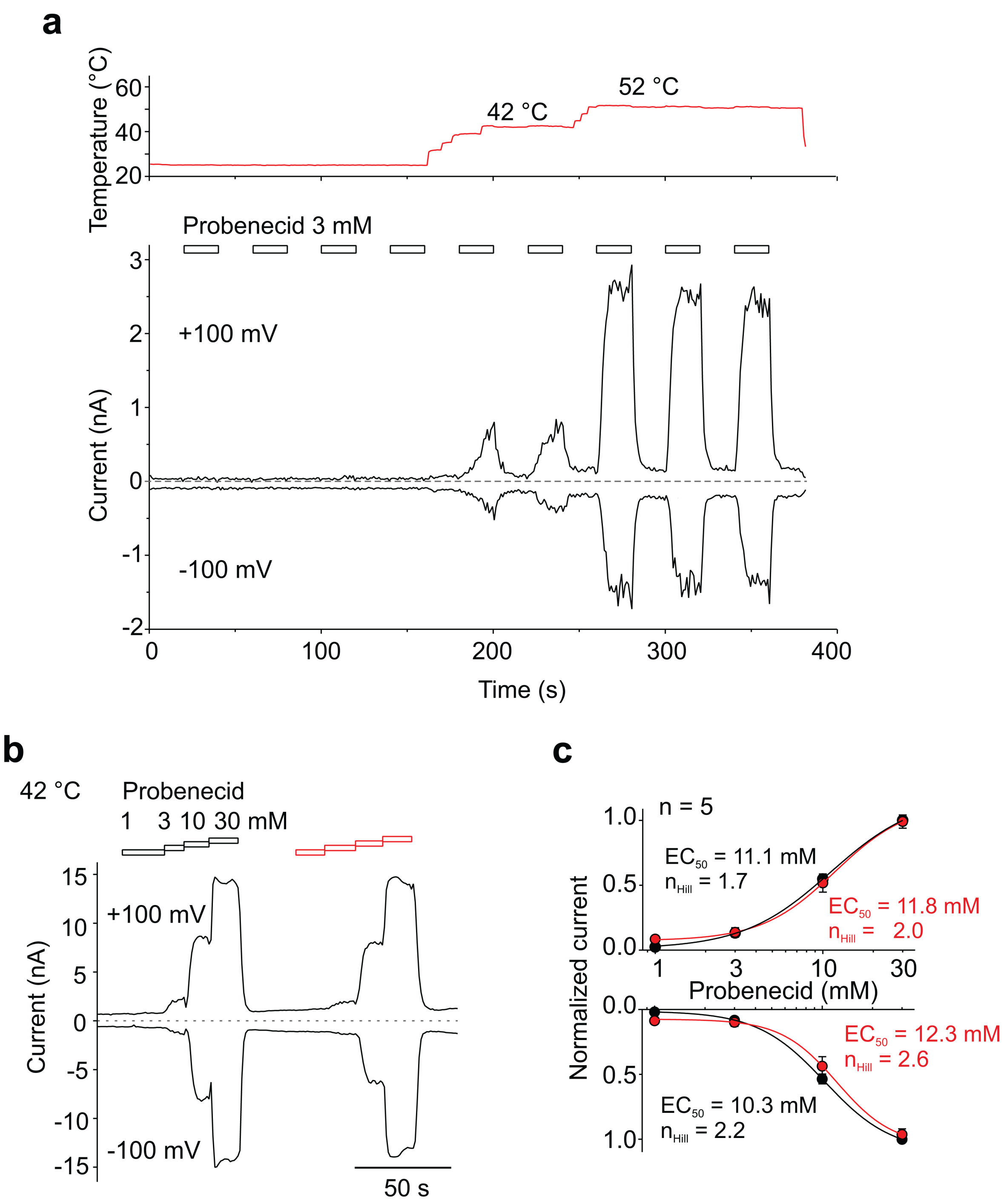

Supplementary Figure 5. Temperature-dependent effects of probenecid on rat TRPV2 currents in HEK293T cells. a Representative whole-cell current recording from a HEK293T cell expressing TRPV2. Currents were elicited by voltage ramps from -100 mV to +100 mV (0.4 V/s) delivered every 1 s from a holding potential of 0 mV. Probenecid 3 mM (marked as white horizontal bars) was applied repeatedly to the cell at temperatures indicated above. **b** Currents induced by probenecid applied sequentially at concentrations 1, 3, 10, and 30 mM twice in succession, recorded at 42 °C. **c** Lack of use-dependent effects of probenecid as measured twice in succession (black and red lines and symbols) from recordings such as shown in (**b**). Data were normalized to the maximal response produced by 30 mM probenecid at +100 mV or -100 mV and fitted by concentration-response curves. Data are shown as means ± SEM from five independent experiments recorded in extra- and intracellular Ca^{2+}-free solutions. Source data are provided as a Source Data file.

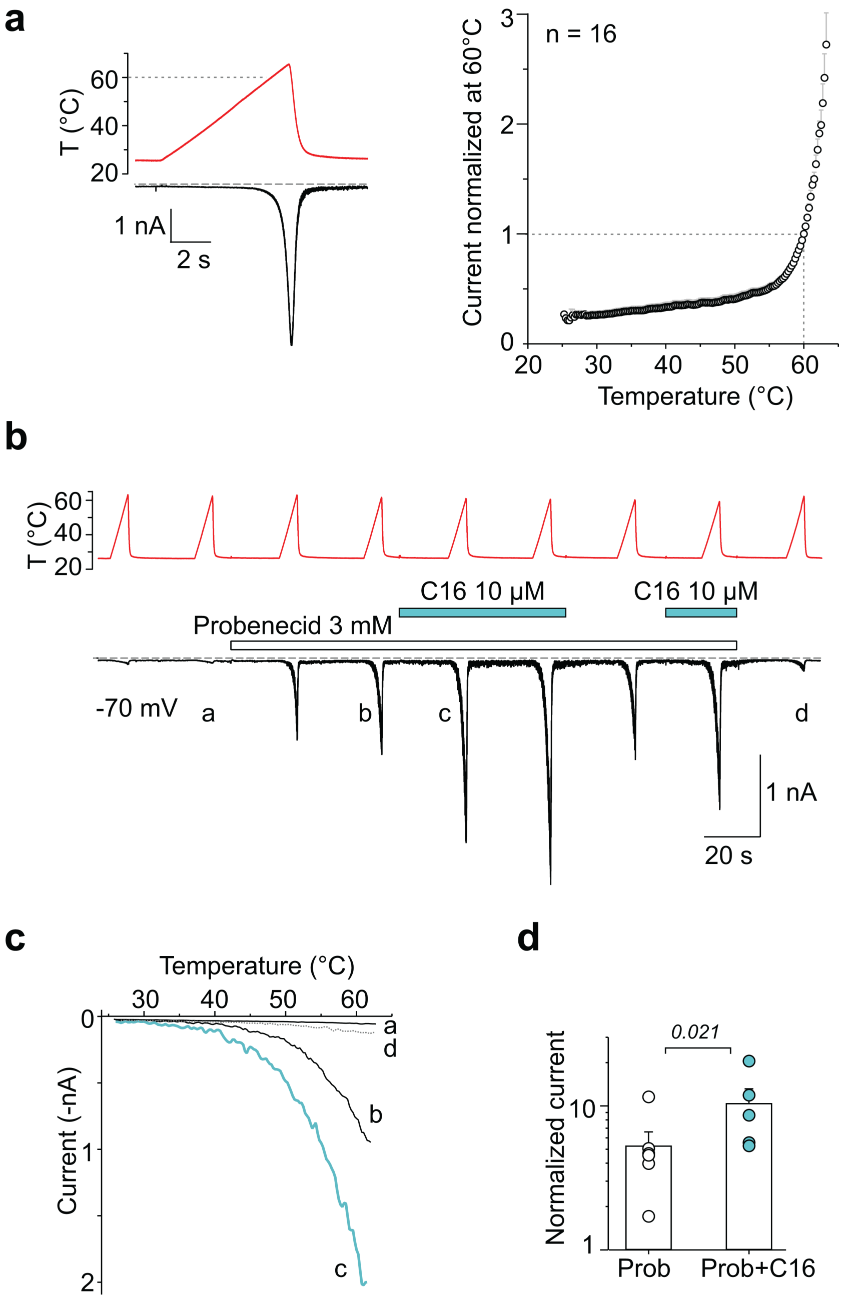

Supplementary Figure 6. Synergistic effect of C16 and probenecid on rat TRPV2 heat-induced currents in HEK293T cells. a Temperature ramps from 25 °C to ~60-62 °C (5 °C/s) at a holding potential of -70 mV, trigger inward currents with a threshold of approximately 55 °C. Average heat-induced currents measured from 16 cells expressing TRPV2, normalized at 60 °C. Data are shown as mean and uni-directional gray bars indicating SEM. **b** Representative whole-cell currents evoked by heat stimuli in the absence or presence of probenecid and C16, applied at -70 mV. **c** Current-temperature relationship of the heat ramp-evoked responses a, b, c and d in (**b**). **d** Statistical summary of the currents induced by probenecid (Prob) and probenecid with C16 (Prob+C16), measured at -70 mV in (**b, c**), with a 2.1 ± 0.3 – fold increase in currents induced by probenecid in the presence of C16. Currents evoked by probenecid (response b) and C16 (response c) were normalized to currents measured in control extracellular solution (response a) and compared at 50 °C. Data are shown as means ± SEM from six (Prob) and five (Prob+C16) independent experiments recorded in extra- and intracellular Ca^{2+}-free solutions. *P* value is given in italics (Student's two-sided unpaired t-test). Source data are provided as a Source Data file.

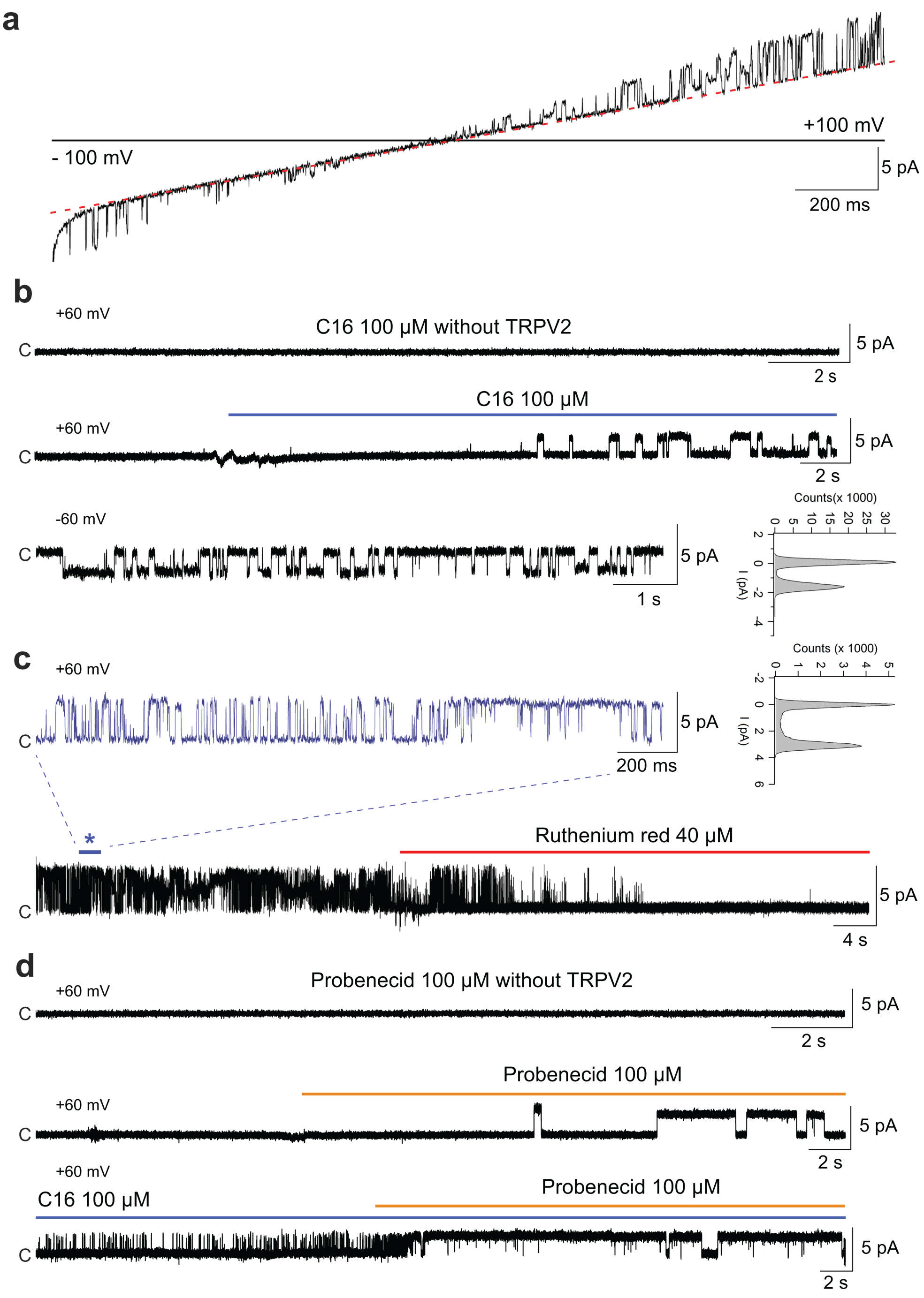

Supplementary Figure 7. C16 and probenecid activates purified rat TRPV2. a As shown in experiments measuring ramp currents (-100 to +100 mV in 2 s), rat TRPV2 responded to C16 (100 µM) with currents at both negative and positive test potentials (black trace). Red dotted line shows zero channel current level. **b** At a steady-state holding potential of +60 mV or -60 mV , distinct single-channel openings were observed in bilayers with (n = 4 independent experiments) but not without (n = 20 independent experiments) TRPV2 when exposed to C16. **c** The TRP channel blocker ruthenium red inhibited C16-evoked TRPV2 currents (n = 4 independent experiments). The blue trace corresponds to events marked with asterisk and is displayed at an expanded timescale. Shown are also the corresponding amplitude histograms. **d** Probenecid alone and in combination with C16 activated TRPV2 (n = 2 independent experiments) but alone had no effect on empty bilayers at a holding potential of +60 mV (n = 4 independent experiments). Purified TRPV2 was reconstituted into planar lipid bilayers and channel currents were recorded with the patch-clamp technique in a symmetrical K^+ solution at room temperature (c = closed channel state, upward or downward deflections = open channel state).

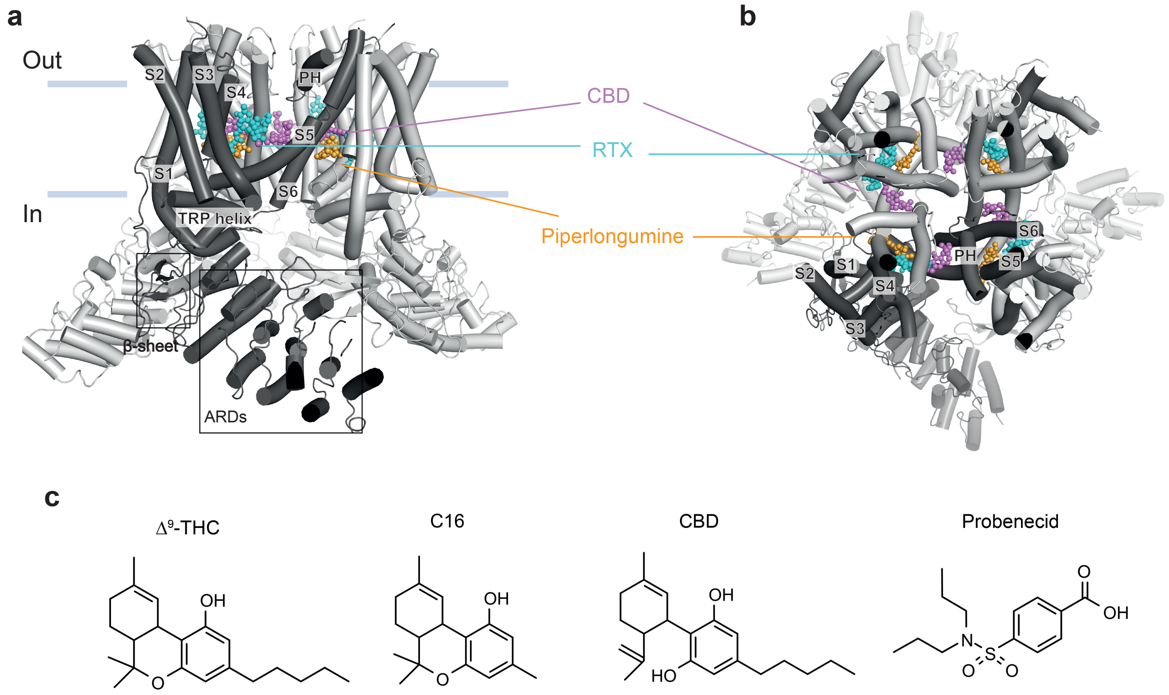

Supplementary Figure 8. Ligand-binding sites in TRPV2. a, b The TRPV2 binding positions of the agonists cannabidiol (CBD, PDB-ID: 6U88)[1] and Resiniferatoxin (RTX, PDB-ID: 6BWJ)[2] as well as the antagonist piperlongumine (PLG, PDB-ID: 6WKN)[3], shown in the membrane plane (**a**) or from the extracellular side (**b**). One subunit of TRPV2 is displayed in dark grey and the remaining three subunits in light grey. Ligands are colored differently and shown as spheres. **c** Shown are the chemical structures of the phytocannabinoids Δ^9-THC (Δ^9-tetrahydrocannabinol), C16 (Δ^9-tetrahydrocannabiorcol) and CBD (cannabidiol) and the non-cannabinoid probenecid, all of which activate TRPV2. In contrast to cannabidiol, Δ^9-THC and C16 are ABC-tricyclic terpenoid compounds with a benzopyran moiety.

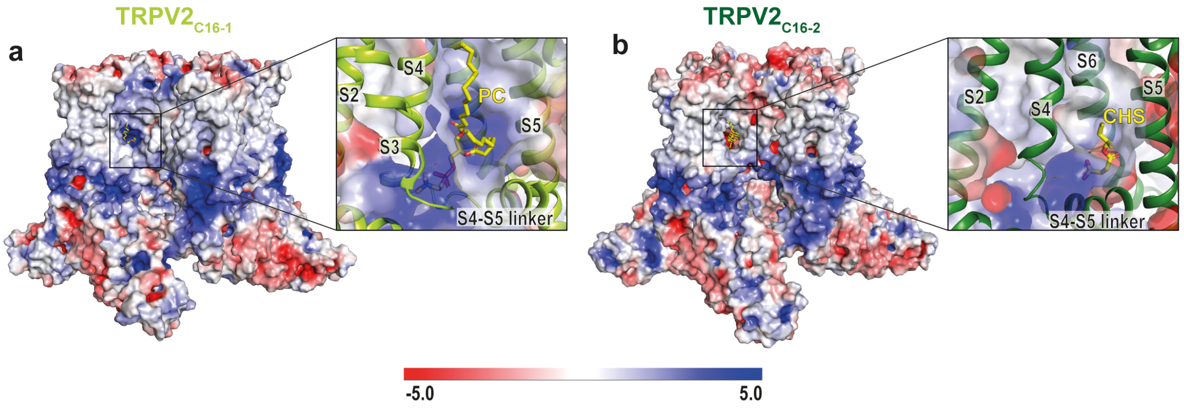

Supplementary Figure 9. Surface charge analysis of TRPV2$_{C16-1}$ and TRPV2$_{C16-2}$. Overall structures with close-views of the vanilloid pockets. **a** TRPV2$_{C16-1}$ with PC (yellow sticks) in the vanilloid pocket. **b** TRPV2$_{C16-2}$ with CHS (yellow sticks) in the vanilloid pocket.

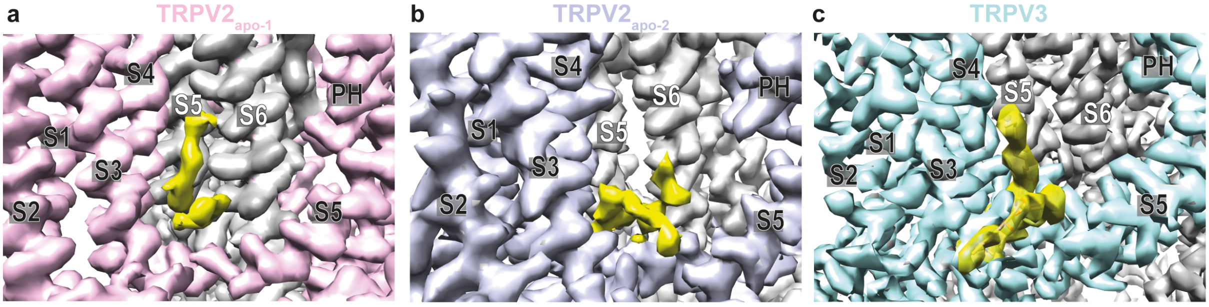

Supplementary Figure 10. Lipids in the vanilloid pocket. Cryo-EM density in the vanilloid pocket of $TRPV2_{apo-1}$ (**a**), $TRPV2_{apo-2}$ (**b**) and TRPV3 (**c**). Lipid density is colored yellow. Density for one subunit of each structure is colored grey, while the adjacent subunit is colored pink ($TRPV2_{apo-1}$, EMD-20677)[1], light blue ($TRPV2_{apo-2}$, EMD-20678)[1] and cyan (TRPV3, EMD-0882)[4].

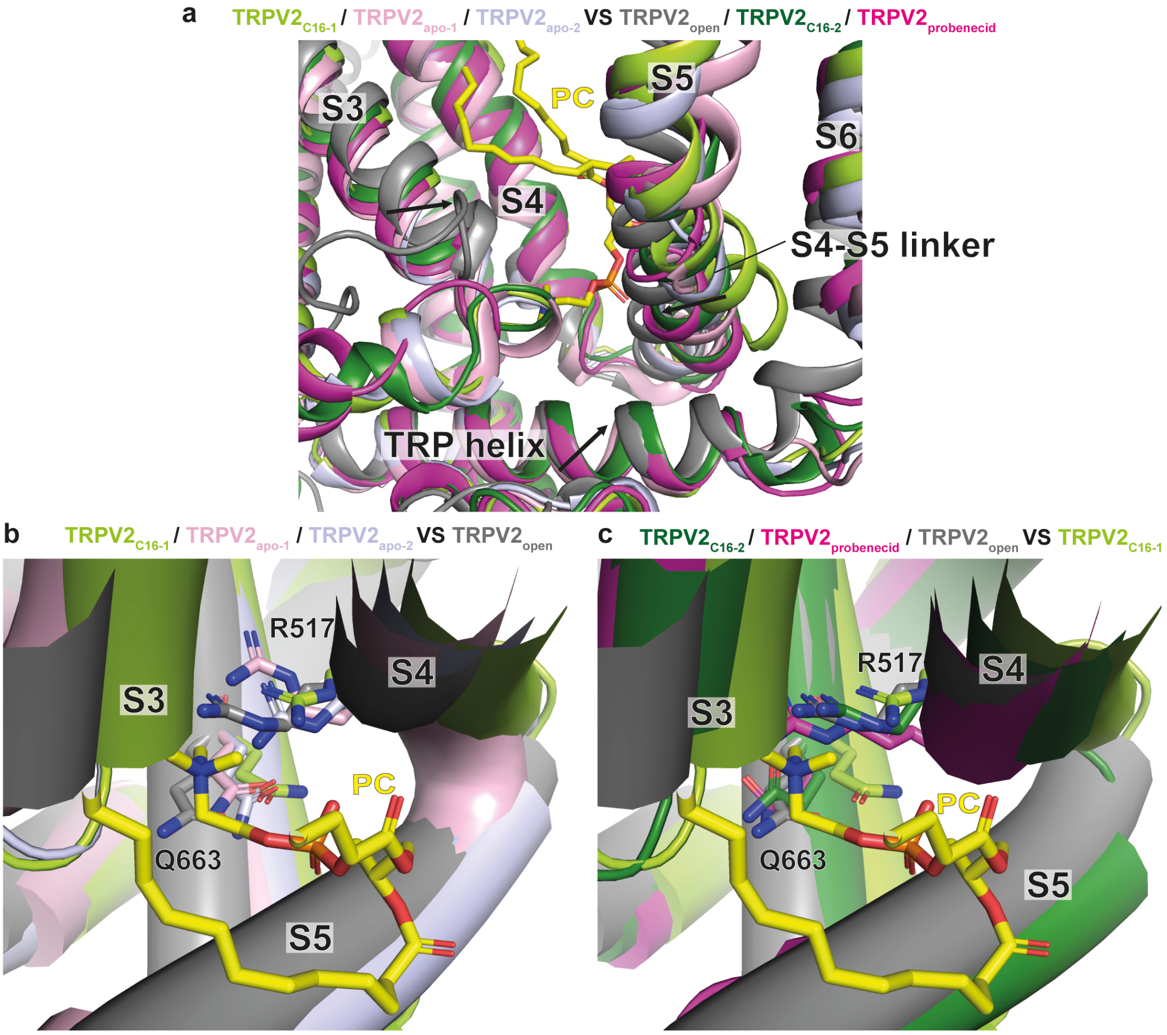

Supplementary Figure 11. The location of the proposed lipid in TRPV2. a Overlay of $TRPV2_{C16\text{-}1}$ (light green), $TRPV2_{apo\text{-}1}$ (PDB-ID: 6U84, pink)[1], $TRPV2_{apo\text{-}2}$ (PDB-ID: 6U86, light blue)[1], which are able to accommodate lipid, as well as of $TRPV2_{open}$ (PDB-ID: 6BO4, grey)[5], $TRPV2_{C16\text{-}2}$ (dark green) and $TRPV2_{probenecid}$ (magenta), which are not harboring lipid. Arrows indicate the helix shifts in TRPV2 structures with and without lipid. **b** Overlay of TRPV2s with lipid present and with $TRPV2_{open}$ as a reference structure without lipid, showing movements of specific residues. **c** Overlay of TRPV2s without lipid present and with $TRPV2_{C16\text{-}1}$ as a reference structure with lipid, showing movements of specific residues. The alignments are based on a single subunit.

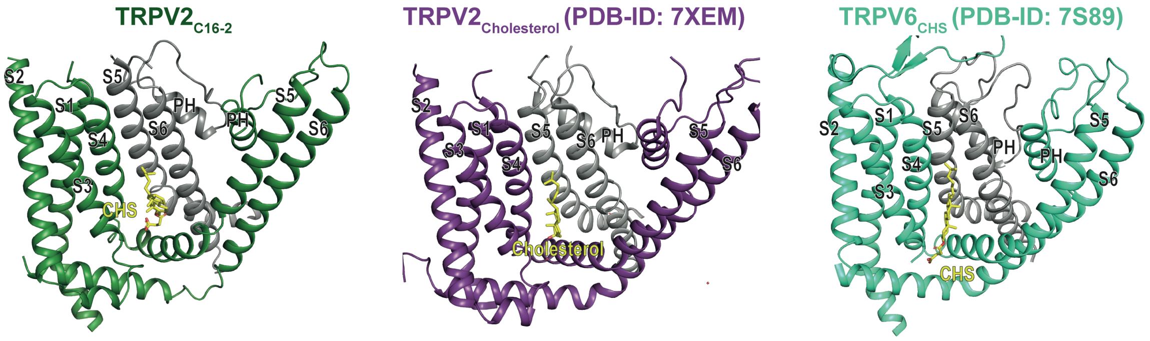

Supplementary Figure 12. The location of the proposed CHS-like lipid in vanilloid pocket of TRPV family members. Comparisons of CHS-like lipid discovered in vanilloid pocket of C16-treated TRPV2 with available CHS-modelled in TRPV2 and TRPV6. An adjacent subunit (S5-PH-S6) is shown in grey.

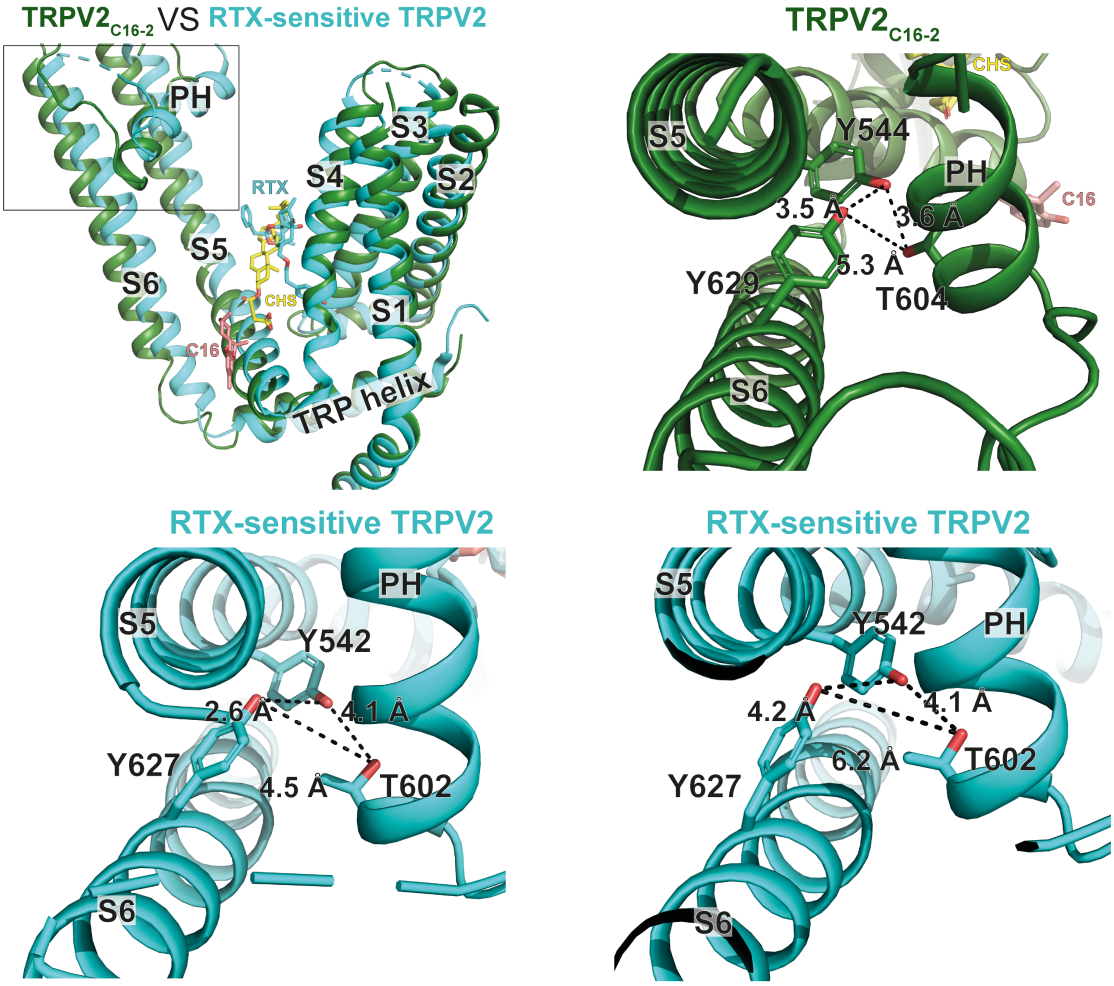

Supplementary Figure 13. The hydrogen-bond triad network. Subunit overlay of $TRPV2_{C16\text{-}2}$ (dark green) and $TRPV2_{RTX}$ (cyan). Resiniferatoxin (RTX), CHS and C16 are shown in cyan-, yellow- and pink-colored sticks, respectively. Small panels show hydrogen triad networks in TRPV2 structures in the presence of C16 or RTX (PDB-ID: 6OO7)[6]. Two types of hydrogen-bond triads are detected in an RTX-sensitive TRPV2 form treated by RTX. The residues Y544, T604 and Y629 forming the hydrogen-bond triad are labelled and displayed in sticks.

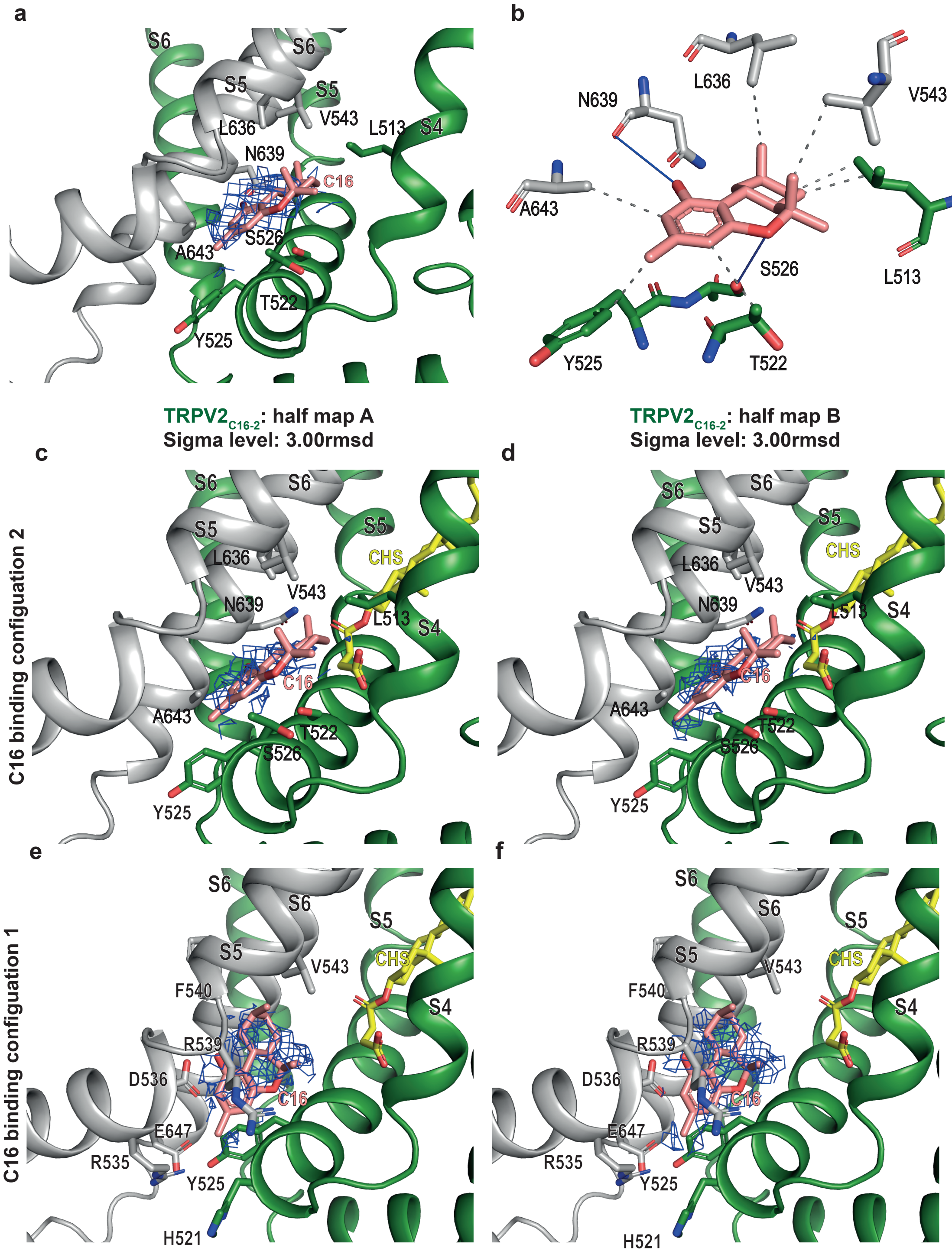

Supplementary Figure 14. Alternative C16 pose in TRPV2$_{C16\text{-}2}$. **a** Model of TRPV2$_{C16\text{-}2}$ (with one subunit colored in dark green and the adjacent in grey) with C16 shown as pink sticks and the corresponding cryo-EM density as blue mesh (3.0 σ, at which protein sidechains are distinguishable). **b** Hydrogen and hydrophobic interactions between C16 (pink sticks) and nearby residues in TRPV2C$_{16\text{-}2}$. **c-f** Ligand cryo-density in both half-maps for each possible binding configuration (3.0 σ).

.

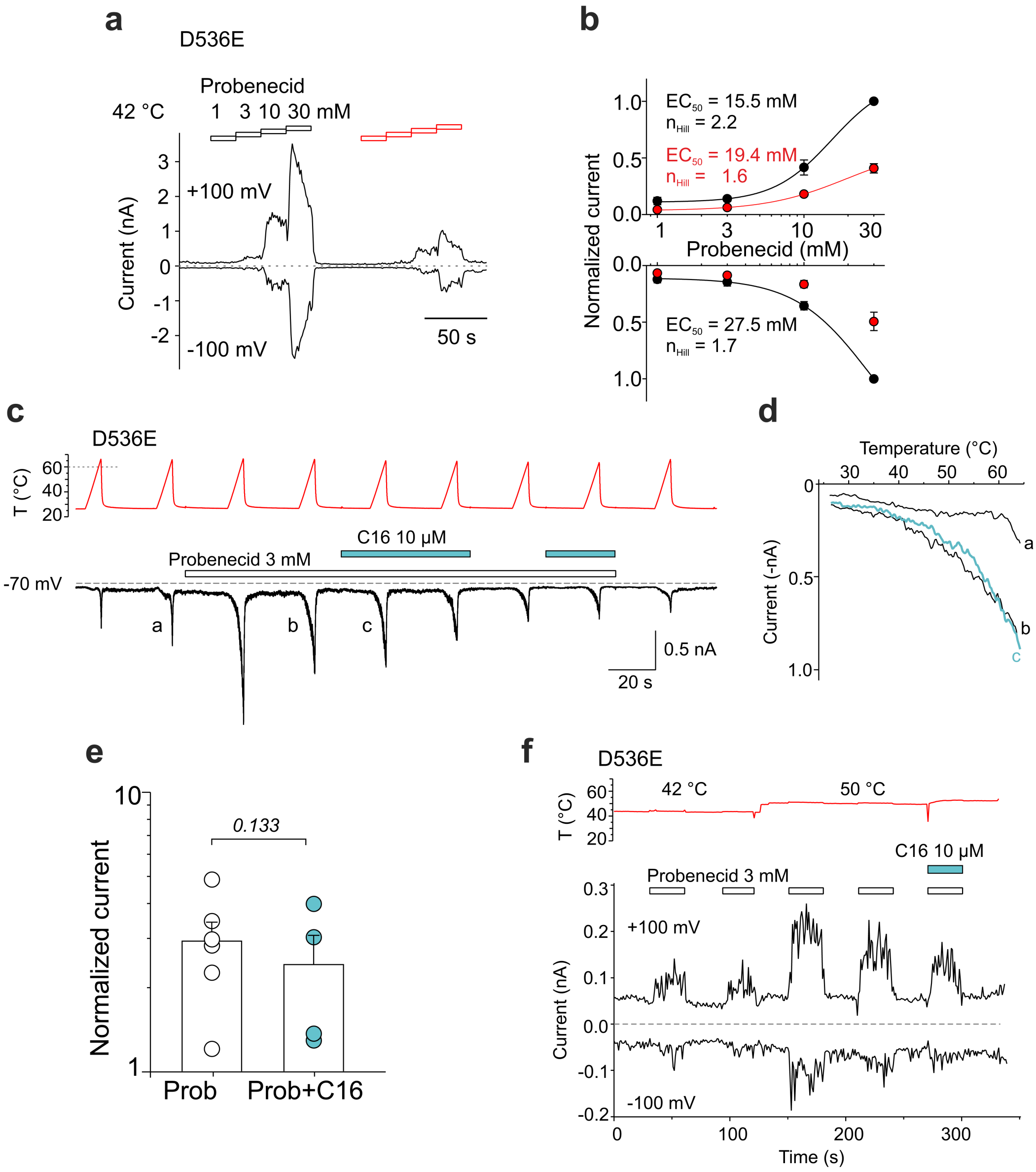

Supplementary Figure 15. Effects of C16, probenecid and heat on the D536E mutant of rat TRPV2. a Representative whole-cell currents elicited by probenecid applied sequentially at concentrations of 1, 3, 10 and 30 mM, twice in succession (marked by white horizontal bars) at 42 °C. **b** Data were normalized to the maximal response produced by 30 mM probenecid at +100 mV or -100 mV and (except for the 2nd application of the concentration series of probenecid measured at -100 mV) fitted by concentration-response curves. Data are shown as means ± SEM from seven (1st exposure) and four (2nd exposure) separate experiments recorded in extra- and intracellular Ca^{2+}-free solutions. **c** C16 does not poteniate D536E-mediated currents elicited by probenecid. Representative whole-cell currents evoked by heat stimuli in the absence or presence of probenecid (white horizontal bars) and C16 (cyan horizontal bars), applied at -70 mV. **d** Current-temperature relationship of the heat ramp-evoked responses a, b and c in (**c**). **e** Statistical summary of the currents induced by probenecid (Prob) and probenecid with C16 (Prob+C16), measured at -70 mV in (**c, d**). Currents evoked by probenecid (response b) and C16 (response c) were normalized to currents measured in control extracellular solution (response a) and compared at 50 °C. Data are shown as means ± SEM from six (Prob) and four (Prob+C16) independent experiments recorded in extra- and intracellular Ca^{2+}-free solutions. *P* value is given in italics (Student's two-sided unpaired t-test). **f** Currents elicited by voltage ramps from -100 mV to +100 mV induced by probenecid 3 mM (white horizontal bars) and C16 (cyan horizontal bar) applied to the cell at temperatures indicated above. Source data are provided as a Source Data file.

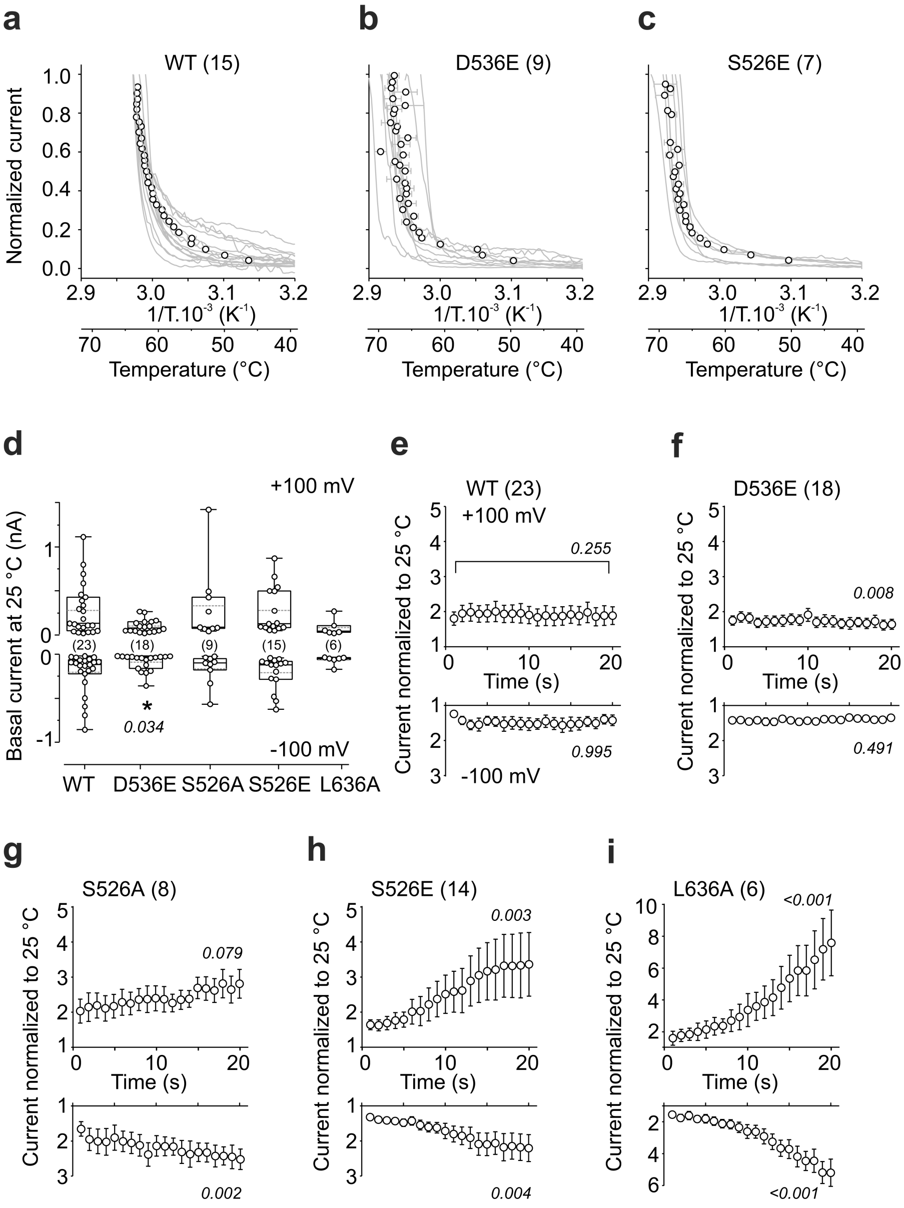

Supplementary Figure 16. Effects of heat on rat TRPV2 mutants. a-c Current-temperature relationships for heat responses in HEK293T cells expressing the indicated constructs of rat TRPV2, normalized to the maximum amplitude, with average values overlaid (empty circles with grey error bars indicating SEM). The number of cells is indicated within parentheses. **d-i** Heat modulation of basal currents. **d** Basal currents at 25 °C were not significantly different in cells expressing wild-type (WT), D536E, S526A, S526E, or L636A mutants at +100 mV (one-way Anova, P = 0.086). At -100 mV, D536E exhibited significantly lower basal currents compared to wild-type (Student's two-sided unpaired t-test, P = 0.034). In the box plot, the horizontal lines indicate the median (solid line) and mean (gray dashed line), the bottom and top edges of the box indicate the interquartile range, and the whiskers represent the maximum and minimum data points. **e-i** The average currents measured at -100 mV and +100 mV during the first 20 s of exposure to 42 °C, normalized to currents at 25 °C. Compared with 25 °C, the currents increased significantly at 42 °C in S526A, S526E and L636A, but not in WT and D536E. P values are given in italics, Student's two-sided paired t-test between the first and last two seconds of the recordings. **i** For L636A, P = 0.0008791 and 0.0000262 at -100 mV and -100 mV, respectively. Data are represented as mean ± SEM from independent experiments, the number of cells is indicated within parentheses, recorded in extra- and intracellular Ca^{2+}-free solutions. Missing error bars are smaller than symbols. Note the doubling in scale on the vertical axis for L636A. Source data are provided as a Source Data file.

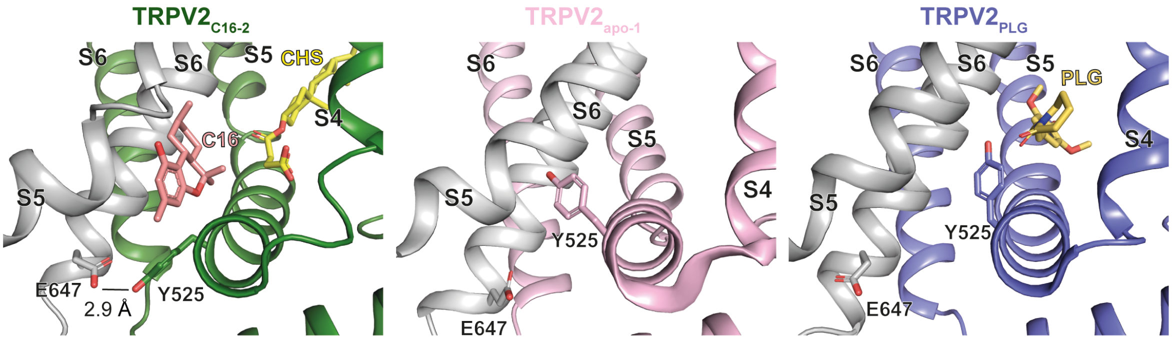

Supplementary Figure 17. E647 and Y525. Close-views of the C16 and PLG interacting sites in TRPV2$_{C16\text{-}2}$ (dark green), TRPV2$_{apo\text{-}1}$ (PDB-ID: 6U84, pink)[1] and TRPV2$_{PLG}$ (PDB-ID: 6WKN, dark blue)[3]. Residues of interest are labelled and shown as sticks. C16 is shown as pink sticks, and CHS and piperlongumine (PLG) are displayed as yellow sticks. An adjacent subunit (S5-S6) is shown in grey.

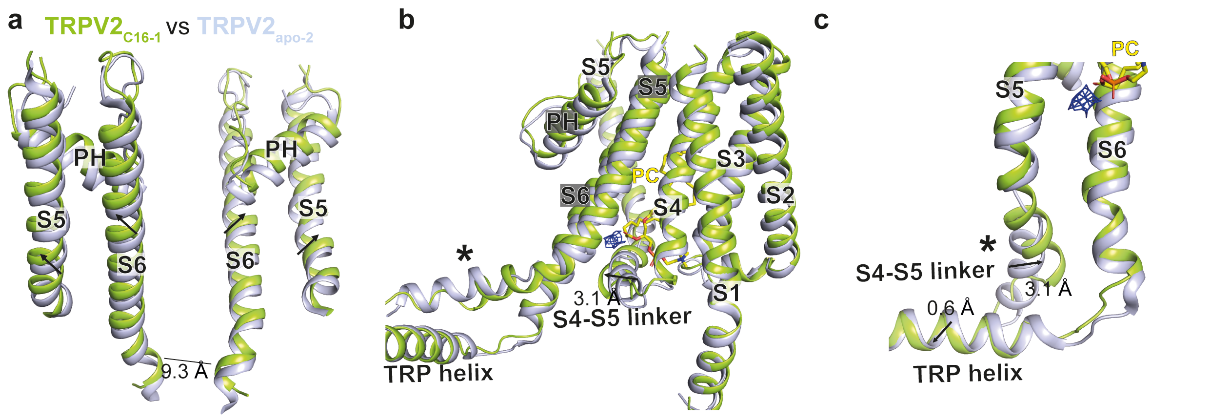

Supplementary Figure 18. Membrane view comparisons of TRPV2$_{C16-1}$ (light green) and TRPV2$_{apo-2}$ (light blue). The TRPV2$_{apo-2}$ structure represents PBD-ID: 6U86[1]. The structures have been aligned globally. **a** Opposite pore forming subunits. **b** The vanilloid pocket shown by a single subunit (S1-S5) and an adjacent subunit (S5-PH-S6-TRP helix). **c** View from the intracellular side. The putative C16 binding site in TRPV2$_{C16-1}$ is shown as blue mesh with contour level at 3.0 σ. Yellow sticks represent the bound lipid molecule.

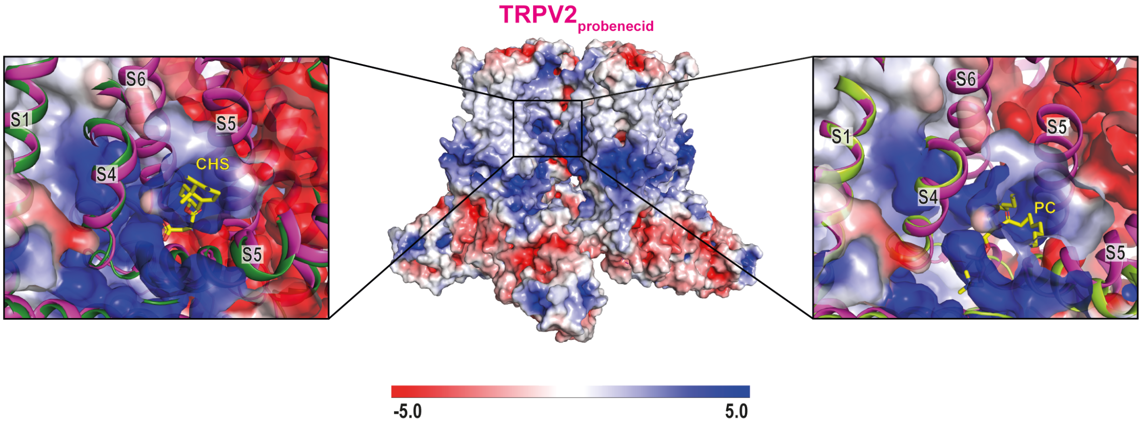

Supplementary Figure 19. Surface charge analysis of TRPV2$_{probenecid}$. Overall structure (center) with close-views of the vanilloid pocket, which lacks ordered lipids in contrast to TRPV2$_{C16-2}$ (left) and TRPV2$_{C16-1}$ (right). Both the CHS-like and the bound lipids are displayed as yellow sticks. The alignments of TRPV2$_{C16-2}$ vs TRPV2$_{probenecid}$ (left) and TRPV2$_{C16-1}$ vs TRPV2$_{probenecid}$ (right) are based on the S1-S4 of one subunit.

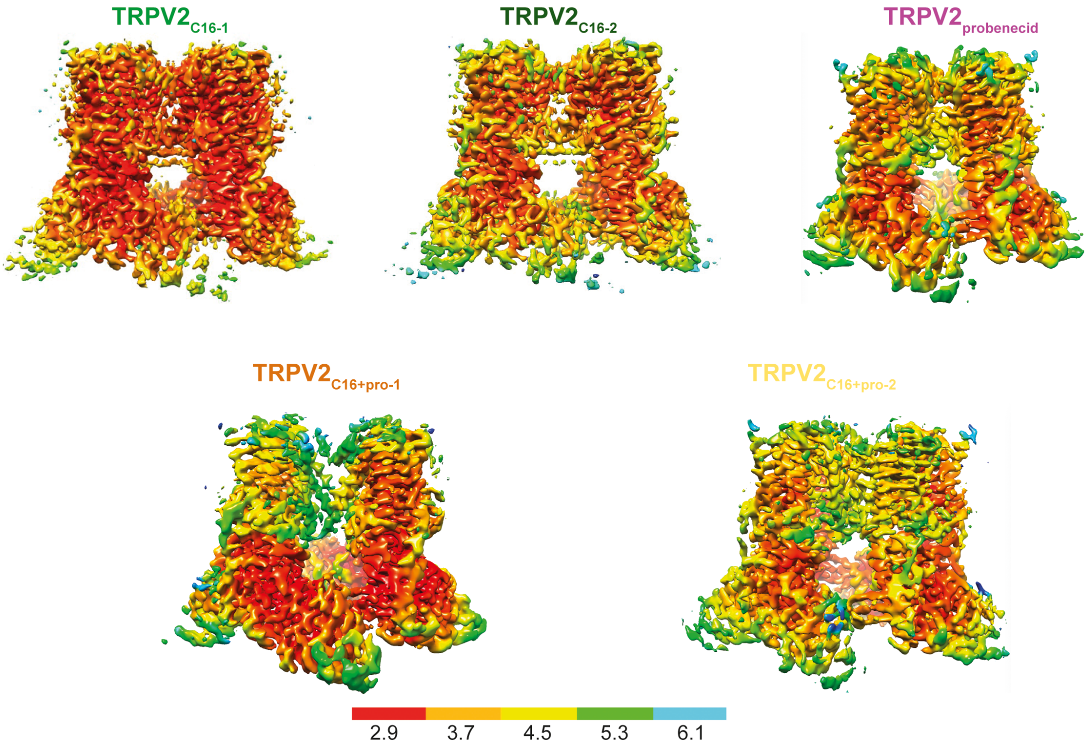

Supplementary Figure 20. Local resolution density maps. Density maps for all ligand-treated TRPV2 states colored by local resolution, showing relatively low resolution at the pore region, especially at the SF and the LG.

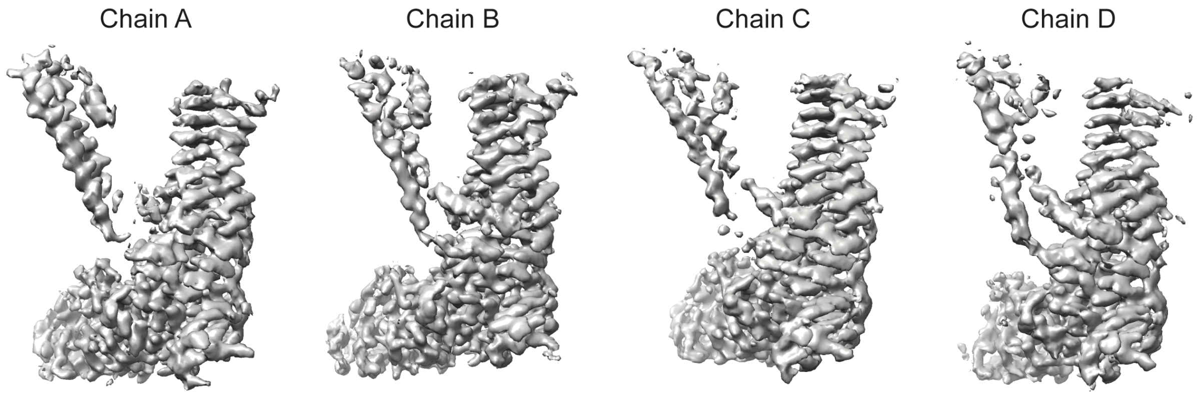

Supplementary Figure 21. Representative densities of each chain from the $TRPV2_{C16+pro-1}$ cryo-EM map, contoured at level 0.25 in Chimera.

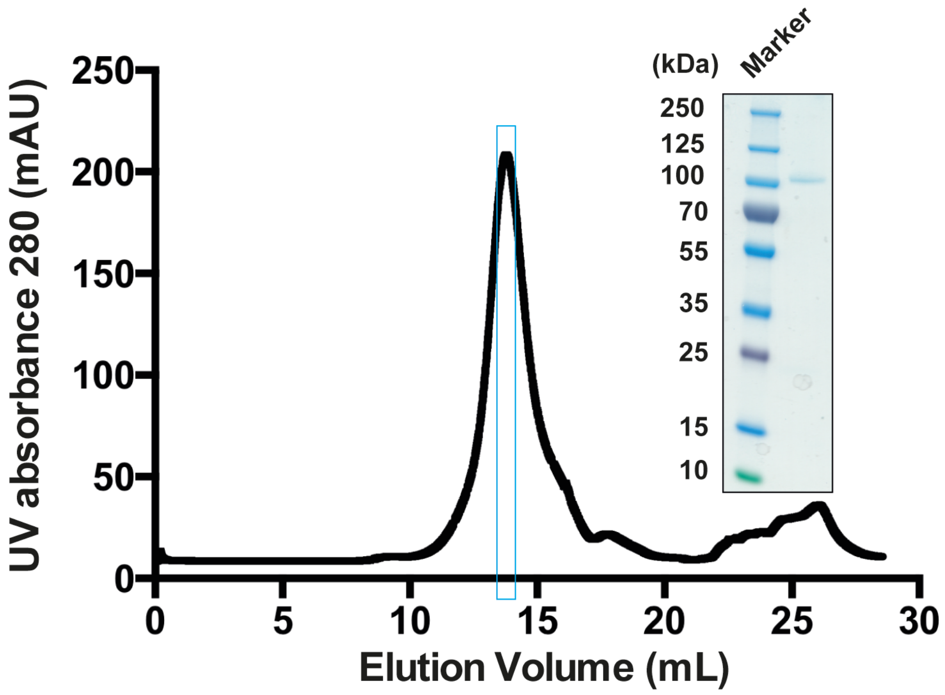

Supplementary Figure 22. The quality of purified protein. Size-exclusion chromatography profile using a Superose 6 column of purified TRPV2 and Coomassie stained SDS-PAGE of samples from the indicated (blue box in the SEC profile) top fraction for freezing (n = 10 independent experiments). The uncropped gel is supplied at the end of the Supplementary Information file.

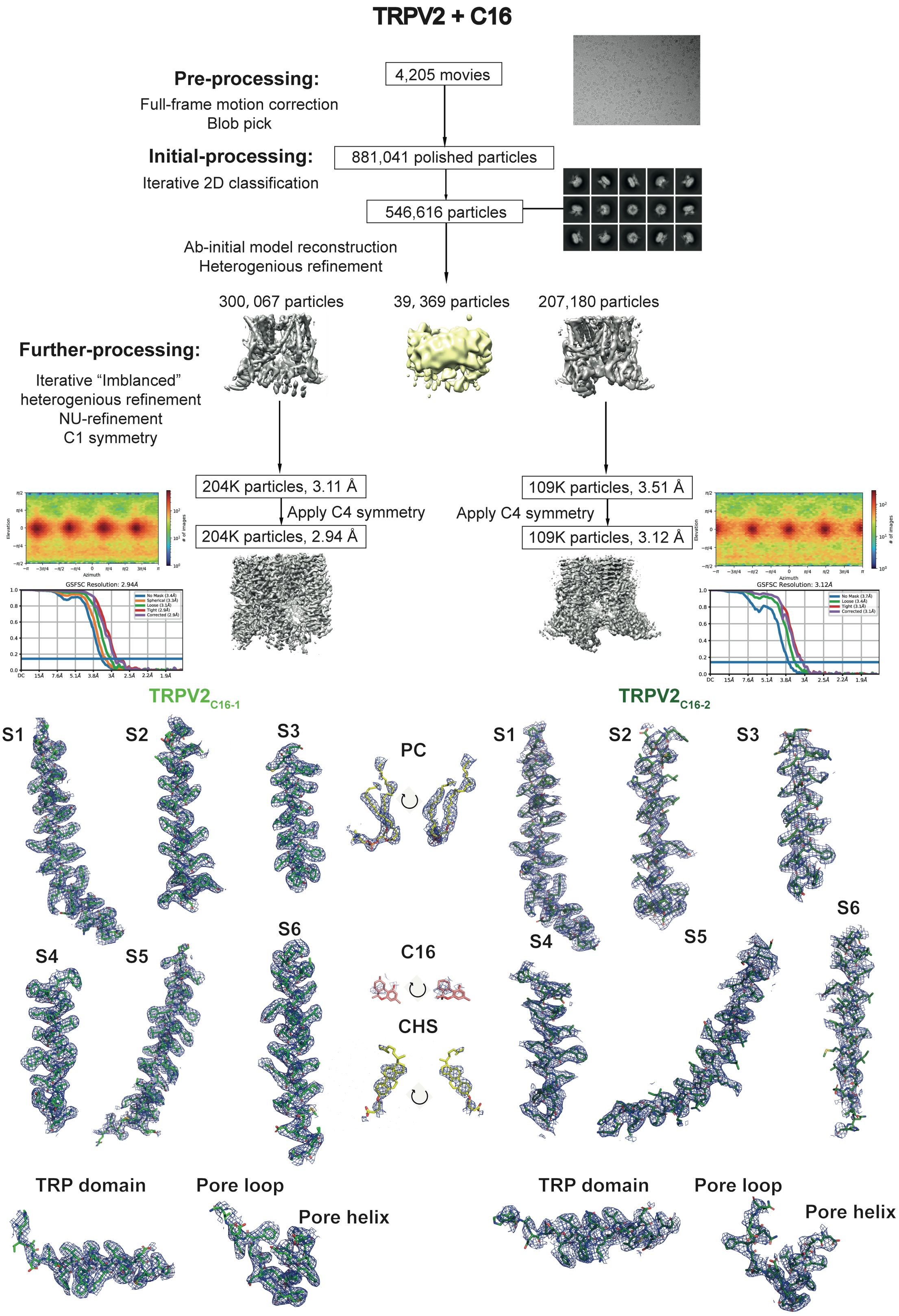

Supplementary Figure 23. C16-treated TRPV2 cryo-EM data processing pipeline and quality of the cryo-EM density. Workflow for 3D reconstruction resulting in two distinct TRPV2 structures. Representative densities from the cryo-EM map of TRPV2$_{C16-1}$ and TRPV2$_{C16-2}$. Densities of PC in TRPV2$_{C16-1}$ or CHS and C16 in TRPV2$_{C16-2}$ are presented with two views. Densities are contoured at 5.0 σ.

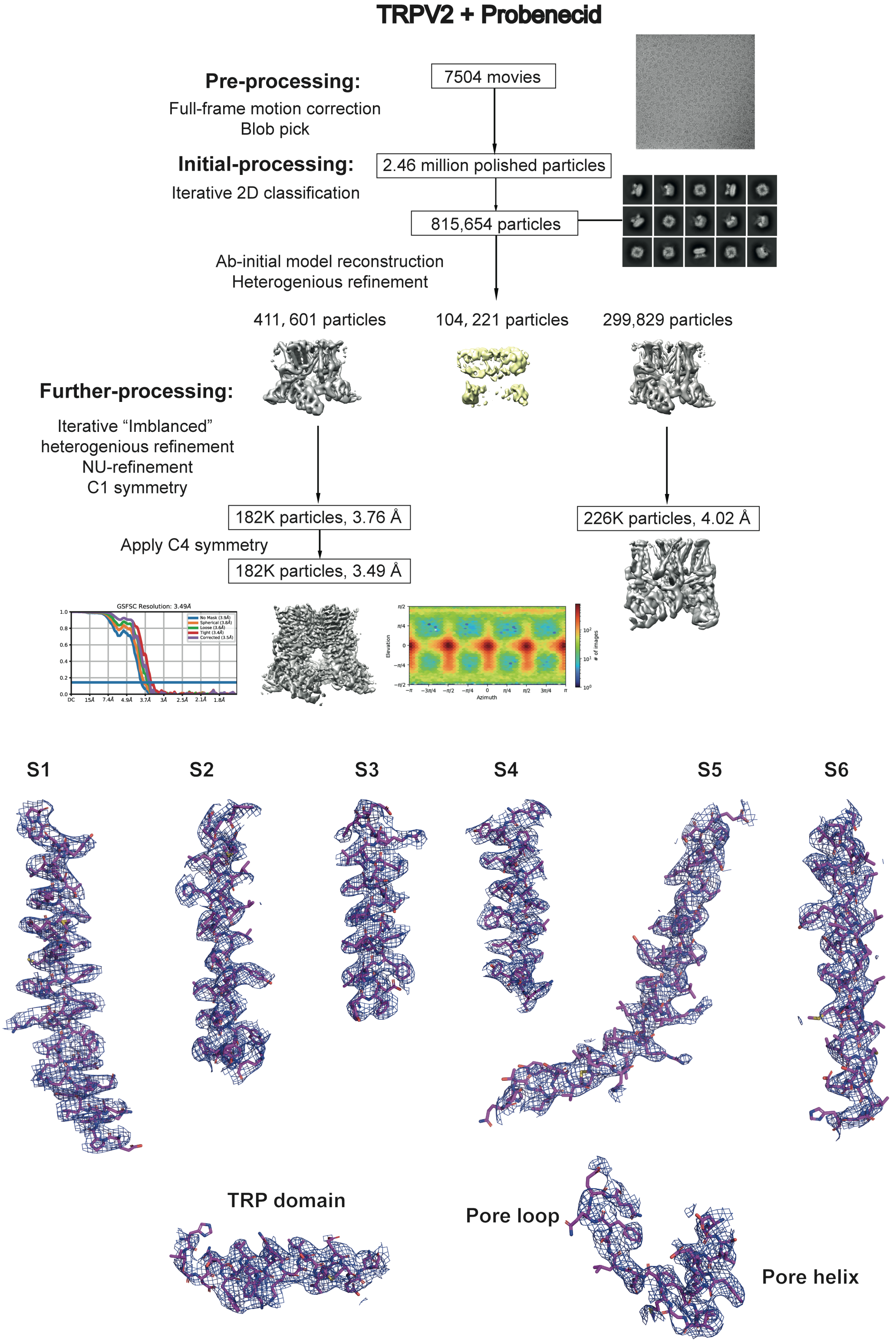

Supplementary Figure 24. Probenecid-treated TRPV2 cryo-EM data processing pipeline and quality of the cryo-EM density. Workflow for 3D reconstruction resulting in two distinct TRPV2 structures. Representative densities from the cryo-EM map of TRPV2$_{probenecid}$. Densities are contoured at 5.0 σ.

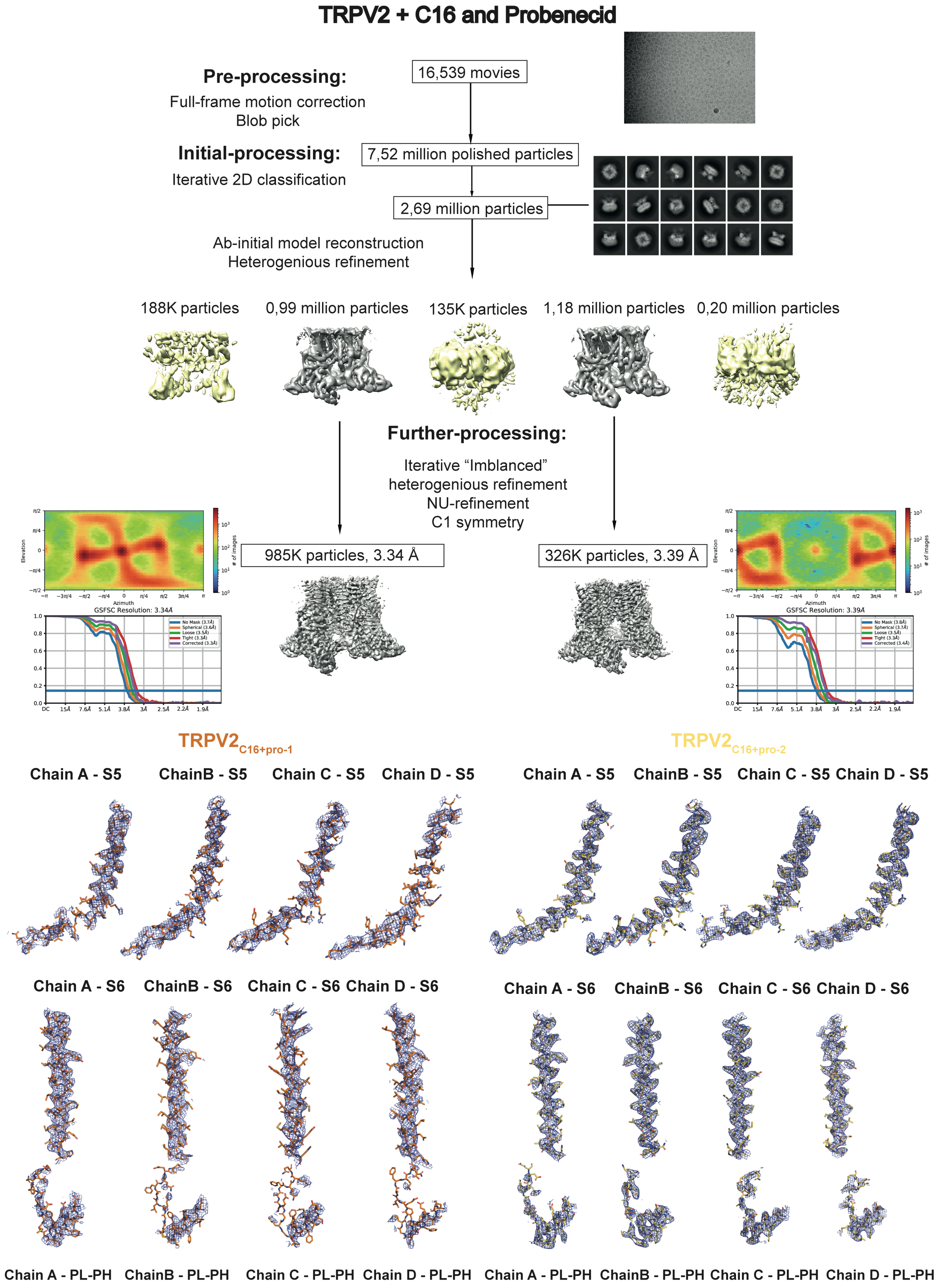

Supplementary Figure 25. C16 plus Probenecid-treated TRPV2 cryo-EM data processing pipeline and quality of the cryo-EM density. Workflow for 3D reconstruction resulting in two distinct TRPV2 structures, $TRPV2_{C16+pro-1}$ and $TRPV2_{C16+pro-2}$. Representative densities for the pore part of each chain from the cryo-EM map of $TRPV2_{C16+pro-1}$ and $TRPV2_{C16+pro-2}$. Densities are contoured at 5.0 σ.

Supplementary Table 1. Root mean square deviation (Å) overview for tetramer comparisons of structures (or parts thereof).

Structure	RMSD values (Å)		
	**Global**	**S1-S6**	**S1-S4**
$TRPV2_{C16\text{-}2}$ vs $TRPV2_{C16\text{-}1}$	2.5	2.3	2.2
$TRPV2_{C16\text{-}2}$ vs $TRPV2_{apo\text{-}1}$	2.6	1.8	1.7
$TRPV2_{C16\text{-}2}$ vs $TRPV2_{PLG}$	1.2	0.8	0.6
$TRPV2_{C16\text{-}1}$ vs $TRPV2_{apo\text{-}2}$	1.4	0.9	0.9
$TRPV2_{probenecid}$ vs $TRPV2_{apo\text{-}1}$	2.1	1.8	1.7

Supplementary Table 2. Data processing, refinement, and model statistics.

Structure	TRPV2$_{C16-1}$	TRPV2$_{C16-2}$	TRPV2$_{probenecid}$	TRPV2$_{C16+pro-1}$	TRPV2$_{C16+pro-2}$
EMDB accession code	EMD-14745	EMD-14746	EMD-14747	EMD-14749	EMD-14748
PDB accession code	7ZJD	7ZJE	7ZJG	7ZJI	7ZJH
**Data collection and processing**					
Magnification			105k		
Voltage (kV)			300		
Electron exposure (e^-/Å^2)	48.4	48.4	57	50	50
Defocus range (µm)			0.7 - 2.7		
Pixel size (Å)	0.8464	0.8464	0.82	0.8464	0.8464
Symmetry imposed	C4	C4	C4	C1	C1
Initial particle images (no.)	300K	207K	412K	985K	1180K
Final particle images (no.)	204K	109K	182K	985K	326K
Map resolution (Å)	2.94	3.12	3.49	3.34	3.39
FSC threshold	0.143	0.143	0.143	0.143	0.143
**Refinement**					
Initial model used (PDB ID)	6U86	6U84	6U84	6U84	6U84
**Model composition (monomer)**					
Protein residues	2388	2380	2396	2384	2344
Nonhydrogen atoms	19360	19360	19532	19303	18900
**R.m.s. deviations**					
Bond lengths (Å)	0.004	0.003	0.002	0.003	0.003
Bond angles (°)	1.026	0.593	0.713	0.746	0.711
CC mask	0.82	0.79	0.73	0.78	0.81
**Validation**					
MolProbity score	1.88	2.03	1.99	2.05	2.01
Clashscore	7.06	4.40	9.49	8.5	5.55
Poor rotamers (%)	0.00	2.74	0.19	1.39	2.05
**Ramachandran plot**					
Favoured (%)	91.98	92.25	92.05	92.18	92.32
Allowed (%)	7.85	7.41	7.61	7.61	7.42
Disallowed (%)	0.17	0.34	0.34	0.21	0.26

References

1. Pumroy, R.A., *et al.* Molecular mechanism of TRPV2 channel modulation by cannabidiol. *Elife* **8** (2019).
2. Zubcevic, L., Le, S., Yang, H. & Lee, S.Y. Conformational plasticity in the selectivity filter of the TRPV2 ion channel. *Nat Struct Mol Biol* **25**, 405-415 (2018).
3. Conde, J., *et al.* Allosteric Antagonist Modulation of TRPV2 by Piperlongumine Impairs Glioblastoma Progression. *ACS Cent Sci* **7**, 868-881 (2021).
4. Shimada, H., *et al.* The structure of lipid nanodisc-reconstituted TRPV3 reveals the gating mechanism. *Nat Struct Mol Biol* **27**, 645-652 (2020).
5. Dosey, T.L., *et al.* Structures of TRPV2 in distinct conformations provide insight into role of the pore turret. *Nat Struct Mol Biol* **26**, 40-49 (2019).
6. Zubcevic, L., Hsu, A.L., Borgnia, M.J. & Lee, S.Y. Symmetry transitions during gating of the TRPV2 ion channel in lipid membranes. *Elife* **8** (2019).

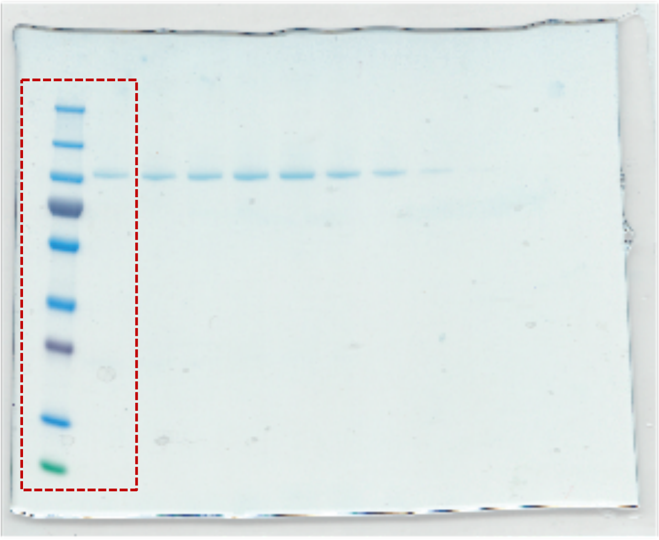

Uncropped SDS-PAGE gel with marked area displayed in Supplementary Figure 22.
