# Supplementary material for: Cannabinoid non-cannabidiol site modulation of TRPV2 structure and function
Source: Nat Commun. 2022 Dec 5;13:7483. doi: 10.1038/s41467-022-35163-y (PMC9722916; doi:10.1038/s41467-022-35163-y)
Supplement: Supplementary file 3 — Reporting Summary [file 41467_2022_35163_MOESM3_ESM.pdf]

nature portfolio

Corresponding author(s): Peter M Zygmunt

Last updated by author(s): Nov 12, 2022

Reporting Summary

Nature Portfolio wishes to improve the reproducibility of the work that we publish. This form provides structure for consistency and transparency in reporting. For further information on Nature Portfolio policies, see our Editorial Policies and the Editorial Policy Checklist.

Statistics

For all statistical analyses, confirm that the following items are present in the figure legend, table legend, main text, or Methods section.

n/a	Confirmed	
☐	☒	The exact sample size (*n*) for each experimental group/condition, given as a discrete number and unit of measurement
☐	☒	A statement on whether measurements were taken from distinct samples or whether the same sample was measured repeatedly
☐	☒	The statistical test(s) used AND whether they are one- or two-sided<br>*Only common tests should be described solely by name; describe more complex techniques in the Methods section.*
☒	☐	A description of all covariates tested
☐	☒	A description of any assumptions or corrections, such as tests of normality and adjustment for multiple comparisons
☐	☒	A full description of the statistical parameters including central tendency (e.g. means) or other basic estimates (e.g. regression coefficient) AND variation (e.g. standard deviation) or associated estimates of uncertainty (e.g. confidence intervals)
☐	☒	For null hypothesis testing, the test statistic (e.g. *F*, *t*, *r*) with confidence intervals, effect sizes, degrees of freedom and *P* value noted<br>*Give P values as exact values whenever suitable.*
☒	☐	For Bayesian analysis, information on the choice of priors and Markov chain Monte Carlo settings
☒	☐	For hierarchical and complex designs, identification of the appropriate level for tests and full reporting of outcomes
☒	☐	Estimates of effect sizes (e.g. Cohen's *d*, Pearson's *r*), indicating how they were calculated

Our web collection on statistics for biologists contains articles on many of the points above.

Software and code

Policy information about availability of computer code

Data collection	Bilayer and whole-cell patch-clamp data were collected using the Port-a-Patch (Nanion Technologies, Germany) and the signals were acquired with an EPC 10 amplifier (HEKA) and the data acquisition software Patchmaster (v2x 65, HEKA). Whole-cell patch-clamp data were also acquired using an Axopatch 200B amplifier and the data acquisition software pCLAMP/Clampfit 9 and 10 (Molecular Devices, USA). Fluorometric calcium measurements were performed on a FLexstation 3 (Molecular Devices) or OlympusIX70 fluorescence microscopy connected to a digital camera (Hamamatsu). Histamine release was measured using the Histareader 501 at RefLab (Copenhagen, Denmark). Single particle cryo-EM data were collected on Titan Krios electron microscopes with a Falcon3 detector or Gatan K3 detector in counting mode.
Data analysis	Bilayer electrophysiological data were analysed using pCLAMP/Clampfit 9 (Molecular Devices), and Igor Pro (6.0.6.0., Wave Metrics software) and GraphPad Prism 9.1. (GraphPad Software, La Jolla, CA) were used for statistical analysis and drawing of graphs. Whole-cell electrophysiological data were analysed using pCLAMP/Clampfit 9 and 10 (Molecular Devices, USA). SigmaPlot 10 (Systat Software Inc., San Jose, USA) was used for statistical analysis and CorelDraw X7 was used for drawing of graphs. Fluorometric calcium imaging data was analysed in SoftMaxPro (Molecular Devices, USA), Excel (Microsoft, version 15, 2013) and GraphPad Prism 9.1 (GraphPad Software, La Jolla, CA). Cryo-EM data were processed with cryosparc version 2.15.0(https://cryosparc.com). Model building was carried out using USCF Chimera 1.15.0, Coot 0.9.4.1, and Phenix 1.20.1-4487. Pore radius was calculated by hole program (http://www.holeprogram.org/) and profile was plotted using Graph Pad Prism 9. Figures were generated using USCF Chimera 1.15.0 and Pymol 2.5.2.

For manuscripts utilizing custom algorithms or software that are central to the research but not yet described in published literature, software must be made available to editors and reviewers. We strongly encourage code deposition in a community repository (e.g. GitHub). See the Nature Portfolio guidelines for submitting code & software for further information.

Data

Policy information about availability of data

All manuscripts must include a data availability statement. This statement should provide the following information, where applicable:

- Accession codes, unique identifiers, or web links for publicly available datasets
- A description of any restrictions on data availability
- For clinical datasets or third party data, please ensure that the statement adheres to our policy

All data presented in graphs within the Figures as well as cryo-EM data supporting the conclusions drawn in this study are available within this article including Supplementary Information and as a Source Data file. Plasmids of the TRPV2 wild-type and mutants thereof investigated in this study are available from the corresponding authors upon request.

The atomic coordinates and cryo-EM density maps generated in this study have been deposited in the Protein Data Bank and Electron Microscopy Data Bank under the accession codes TRPV2C16-1 (PDB 7ZJD [https://doi.org/10.2210/pdb7ZJD /pdb] and EMD-14745 [https://www.ebi.ac.uk/pdbe/entry/emdb/EMD-14745]), TRPV2C16-2 (PDB 7ZJE [https://doi.org/10.2210/pdb7ZJE /pdb] and EMD-14746 [https://www.ebi.ac.uk/pdbe/entry/emdb/EMD-14746], TRPV2probenecid (PDB 7ZJG [https://doi.org/10.2210/pdb7ZJG /pdb] and EMD-14747 [https://www.ebi.ac.uk/pdbe/entry/emdb/EMD-14747], TRPV2C16+pro-1 (PDB 7ZJI [https://doi.org/10.2210/pdb7ZJI /pdb] and EMD-14749 [https://www.ebi.ac.uk/pdbe/entry/emdb/EMD-14749], TRPV2C16+pro-2 (PDB 7ZJH [https://doi.org/10.2210/pdb7ZJH /pdb] and EMD-4748 [https://www.ebi.ac.uk/pdbe/entry/emdb/EMD-14748].

The atomic coordinates and cryo-EM density maps of previously solved TRPV2 structures used in this study are available in the Protein Data Bank and Electron Microscopy Data Bank under the accession codes TRPV2apo-1 (PDB 6U84 [https://doi.org/10.2210/pdb6U84 /pdb] and EMD-20677 [https://www.ebi.ac.uk/pdbe/entry/emdb/EMD-20677]), TRPV2apo-2 (PDB 6U86 [https://doi.org/10.2210/pdb6U86 /pdb] and EMD-20678 [https://www.ebi.ac.uk/pdbe/entry/emdb/EMD-20678]), TRPV2open (PDB 6BO4 [https://doi.org/10.2210/pdb6BO4 /pdb] and EMD-7118 [https://www.ebi.ac.uk/pdbe/entry/emdb/EMD-7118]), TRPV2CHL (PDB 7XEM [https://doi.org/10.2210/pdb7XEM /pdb] and EMD-33156 [https://www.ebi.ac.uk/pdbe/entry/emdb/EMD-33156]), TRPV6 (PDB 7S89 [https://doi.org/10.2210/pdb7S89 /pdb] and EMD-24891 [https://www.ebi.ac.uk/pdbe/entry/emdb/EMD-24891]), TRPV2CBD-1 (PDB 6U8A [https://doi.org/10.2210/pdb6U8A /pdb] and EMD-20686 [https://www.ebi.ac.uk/pdbe/entry/emdb/EMD-20686]), TRPV2CBD-2 (PDB 6U88 [https://doi.org/10.2210/pdb6U88 /pdb] and EMD-20682 [https://www.ebi.ac.uk/pdbe/entry/emdb/EMD-20682]), TRPV2PLG (PDB 6WKN [https://doi.org/10.2210/pdb6WKN /pdb] and EMD-21705 [https://www.ebi.ac.uk/pdbe/entry/emdb/EMD-21705]), TRPV2RTX-C2 (PDB 6OO7 [https://doi.org/10.2210/pdb6OO7 /pdb] and EMD-20148 [https://www.ebi.ac.uk/pdbe/entry/emdb/EMD-20148]), TRPV2RTX-Crystal (PDB 6BWJ [https://doi.org/10.2210/pdb6BWJ /pdb] and X-ray electron density map [https://edmaps.rcsb.org/dsn6/bw/6bwj/]), TRPV2APB+CBD-active (PDB 7T37 [https://doi.org/10.2210/pdb7T37 /pdb] and EMD-25650 [https://www.ebi.ac.uk/pdbe/entry/emdb/EMD-25650]), TRPV3 (PDB 6LGP [https://doi.org/10.2210/pdb6LGP /pdb] and EMD-0882 [https://www.ebi.ac.uk/pdbe/entry/emdb/EMD-0882]).

Field-specific reporting

Please select the one below that is the best fit for your research. If you are not sure, read the appropriate sections before making your selection.

☒ Life sciences ☐ Behavioural & social sciences ☐ Ecological, evolutionary & environmental sciences

For a reference copy of the document with all sections, see nature.com/documents/nr-reporting-summary-flat.pdf

Life sciences study design

All studies must disclose on these points even when the disclosure is negative.

Sample size	Sample size for bilayer and whole-cell patch-clamp measurements as well as fluorometric calcium imaging was determined based on previous recordings using the same experimental conditions and equipment (Andersson et al Nat Commun 2011, Moparthi et al PNAS 2014, Moparthi et al Sci Rep 2016, , Moparthi & Zygmunt Cell Calcium 2020, Zimova et al. 2018, Sinica et al., 2019, Moparthi et al Nat Commun 2022). Mast cell histamine release sample size was based on published literature and own studies (Xiang et al Allergy 2006). Cryo-EM statistics are provided in the Methods section, Supplementary Table 2 and Supplementary Figures 23-25.
Data exclusions	No data exclusions.
Replication	All attempts at replication were successful in bilayer and whole-cell patch-clamp electrophysiology studies as well as fluorometric calcium imaging and histamine release studies. Bilayer patch-clamp experiments were conducted on separate bilayers and days, and fluorometric calcium imaging studies were performed on separate transfections/expression/cell batches and days. Whole-cell patch clamp experiments were conducted on cells from several independent transfections. Control experiments with wild-type TRPV2 were regularly conducted in order to monitor replication. Rat TRPV2 tagged with GFP-His tag were stably produced and purified at several occasions. Multiple Cryo-EM grids were prepared and data was collected from one selected grid for each state. For the histamine release experiments using RBL cells, five experiments were performed independently, whereas for the CBMCs (primary cells), seven donors were used, independently.
Randomization	No randomization in bilayer patch-clamp experiments as the successful insertion of rat TRPV2 and channel activity is a prerequisite for further analysis. As in our previous studies, protein insertion was indicated by channel activity in response to high voltage (+100 mV), intermittently tested, within 30 min. If no activity appeared the procedure was repeated with new chips and bilayers. TRPV2-mediated responses were confirmed using ruthenium red as channel blocker. As shown in here and in several previous own studies, bilayers without proteins do not respond to temperature, pressure and chemicals (agonists, vehicles etc). For whole-cell patch-clamp experiments, individual cells investigated are randomly chosen by the experimenter from usually > 100 transfected cells in the petri dish. Only one cell per dish is investigated. The chosen cell must be attached to the bottom of petri dish, look “healthy“ and show a fluorescence indicating successful transfection. In

fluorometric calcium imaging experiments, the application of test compounds was repeatedly alternated between the plate-wells (96-well plates or 8-well plates) containing cells of interest. For the histamine release experiments using RBL cells and for the CBMCs (primary cells), in each independent experiment the cell suspension (cell line or donor) were dispersed onto a plate with wells containing substances of interest.

Blinding

No blinding in bilayer patch-clamp experiments as the successful insertion of rat TRPV2 and channel activity is a prerequisite for further analysis. As in our previous studies, protein insertion was indicated by channel activity in response to high voltage (+100 mV), intermittently tested, within 30 min. If no activity appeared the procedure was repeated with new chips and bilayers. TRPV2-mediated responses were confirmed using ruthenium red as channel blocker. As shown in here and in several previous own studies, bilayers without proteins do not respond to temperature, pressure and chemicals (agonists, vehicles etc). A true blinding in fluorometric calcium imaging and whole-cell electrophysiology was not possible. Cells, solutions and the performance of fluorometric calcium imaging experiments were done by laboratory technicians without knowledge of the mechanism of action of test compounds, whereas collected data was analyzed by the investigator. Although, the patch clamp experimenters also performed the transfection procedures, they did not know the exact nature of the mutant at the time of the experiment, which was revealed at the time of data analysis. Thus, they did not know what was predicted. For the histamine release experiments using RBL cells and for the CBMCs (primary cells), investigators were blinded during data collection/analysis and for the final analysis the data was assembled into the appropriate experimental groups.

Reporting for specific materials, systems and methods

We require information from authors about some types of materials, experimental systems and methods used in many studies. Here, indicate whether each material, system or method listed is relevant to your study. If you are not sure if a list item applies to your research, read the appropriate section before selecting a response.

Materials & experimental systems

n/a	Involved in the study
☒	☐ Antibodies
☐	☒ Eukaryotic cell lines
☒	☐ Palaeontology and archaeology
☒	☐ Animals and other organisms
☐	☒ Human research participants
☒	☐ Clinical data
☒	☐ Dual use research of concern

Methods

n/a	Involved in the study
☒	☐ ChIP-seq
☒	☐ Flow cytometry
☒	☐ MRI-based neuroimaging

Eukaryotic cell lines

Policy information about cell lines

Cell line source(s)

HEK293T cells from (ATCC, CRL-3216, Manassas, VA, USA) or (ECACC, UK); hTRPA1- and hTRPV1-HEK293Trex cells (AstraZeneca, Sweden); Pichia Pastoris (SMD1168, Invitrogen); RBL-2H3 cells (ATCC, Manassas, USA). The human mast cell line (HMC-1.2; kindly provided by Dr Joseph H. Butterfield, Mayo Clinic Hospital, Rochester, MN, USA) .

Authentication

No authentication of any cell line used in this study.

Mycoplasma contamination

The HEK293T cells are regularly tested for mycoplasma contamination (with negative results). Cells are regularly replaced by fresh bathes free of mycoplasma.

Commonly misidentified lines
(See ICLAC register)

No commonly misidentified lines were used in this study.

Human research participants

Policy information about studies involving human research participants

Population characteristics

Umbilical cord blood is taken from healthy mothers with no complications in connection to the delivery.

Recruitment

Mothers at the delivery unit are asked if umbilical cord blood can be taken. Informed consent was obtained from all participants.

Ethics oversight

The cord blood collection, since the donors are unidentifiable for the investigators, has been granted exemption from requiring ethics approval (Regional Ethics Review Board in Stockholm).

Note that full information on the approval of the study protocol must also be provided in the manuscript.
